# The tumor suppressive effect and apoptotic mechanism of TRAIL gene‐containing recombinant NDV in TRAIL‐resistant colorectal cancer HT‐29 cells and TRAIL‐nonresistant HCT116 cells, with each cell bearing a mouse model

**DOI:** 10.1002/cam4.6622

**Published:** 2023-10-16

**Authors:** Bo‐Kyoung Jung, Yong Hee An, Sung Hoon Jang, Gyoungah Ryu, Saet‐byel Jung, Seonhee Kim, Cuk‐Seong Kim, Hyun Jang

**Affiliations:** ^1^ Libentech Co. LTD Daejeon Republic of Korea; ^2^ Graduate School of Medical Science, College of medicine Yonsei University Seoul Republic of Korea; ^3^ Department of Physiology & Medical Science, College of Medicine Chungnam National University Daejeon Republic of Korea

**Keywords:** apoptosis, colorectal cancer, viral infection, viral oncology

## Abstract

**Background:**

TRAIL is an anticancer drug that induces cancer cell apoptosis by interacting with death receptors (DRs). However, owing to low cell‐surface expression of DRs, certain colorectal cancer (CRC) cells resist TRAIL‐induced apoptosis. Newcastle disease virus (NDV) infection can elevate DR protein expression in cancer cells, potentially influencing their TRAIL sensitivity. However, the precise mechanism by which NDV infection modulates DR expression and impacts TRAIL sensitivity in cancer cells remains unknown.

**Methods:**

Herein, we developed nonpathogenic NDV VG/GA strain‐based recombinant NDV (rNDV) and TRAIL gene‐containing rNDV (rNDV‐TRAIL). We observed that viral infections lead to increased DR and TRAIL expressions and activate signaling proteins involved in intrinsic and extrinsic apoptosis pathways. Experiments were conducted in vitro using TRAIL‐resistant CRC cells (HT‐29) and nonresistant CRC cells (HCT116) and in vivo using relevant mouse models.

**Results:**

rNDV‐TRAIL was found to exhibit better apoptotic efficacy than rNDV in CRC cells. Notably, rNDV‐TRAIL had the stronger cancer cell‐killing effect in TRAIL‐resistant CRC cells. Western blot analyses showed that both rNDV and rNDV‐TRAIL infections activate signaling proteins involved in the intrinsic and extrinsic apoptotic pathways. Notably, rNDV‐TRAIL promotes concurrent intrinsic and extrinsic signal transduction in both HCT‐116 and HT‐29 cells.

**Conclusions:**

Therefore, rNDV‐TRAIL infection effectively enhances DR expression in DR‐depressed HT‐29 cells. Moreover, the TRAIL protein expressed by rNDV‐TRAIL effectively interacts with DR, leading to enhanced apoptosis in TRAIL‐resistant HT‐29 cells. Therefore, rNDV‐TRAIL has potential as a promising therapeutic approach for treating TRAIL‐resistant cancers.

## Abstract 2

Schematic representation of direct mechanisms of the rNDV‐TRAIL viruses. rNDV containing the TRAIL gene (rNDV‐TRAIL) can stably express the TRAIL gene. Also, rNDV‐TRAIL activates the NF‐кB pathway and induces the secretion of TRAIL. The expressed or induced TRAIL binds to the TRAIL receptor on DR4 or DR5. DR5 promotes TRAIL‐induced apoptotic pathways. The pro‐apoptotic complex is formed. Caspase 8 is cleaved, activates caspase 3, and triggers apoptosis via the extrinsic pathway. Also, caspase 8 induces an intrinsic pathway. The cytochrome C combines with procaspase‐9 to produce apoptosome. Apoptosome triggers caspase 9 followed by the activation of caspase‐3 which leads to ends up to apoptosis.

## 1 INTRODUCTION

Colorectal cancer (CRC) is a malignant tumor that originates from cancer cells in the rectum, the terminal part of the large intestine. Histologically, the large intestine comprises several layers, including the mucosal, submucosal, muscular, and serosa layers. Rectal cancer typically initiates as an adenocarcinoma within the intestinal mucosa.^1^ The etiology of CRC is multifactorial, with genetic factors being just one of the contributing elements.^2^ The process begins with genetically vulnerable normal cells in the colonic mucosa forming polyps in the large intestine, which subsequently undergo malignant transformation, leading to the development of cancerous cells. As the disease advances, the cancer becomes invasive, and over time, it may progress to a metastatic stage, spreading to other organs.^3^ CRC primarily affects individuals over the age of 50. While mortality and morbidity rates have seen a decline owing to the advancements in diagnosis and treatment, the incidence is on the rise in Asian regions, attributed to dietary changes and increased meat consumption. Presently, colonoscopy plays a crucial role in identifying and removing preneoplastic adenomas as well as detecting CRCs at early stages.^4^ Despite the availability of various diagnosis methods, including colonoscopy and other techniques for detecting CRCs, late‐stage detection (Stage III or IV) remains a significant challenge and often leads to difficulties in achieving optimal health outcomes, even when multiple treatment methods are employed simultaneously.^5^

Several intrinsic and engineered oncolytic viruses hold potential for cancer treatment. For over 50 years, numerous scientists have conducted extensive research on utilizing a specific virus for cancer treatment, which was even identified as the causative agent of a human or animal disease.^6^ Oncolytic viruses, such as Imlygic, have proven to be effective cancer therapeutics for melanoma and were commercialized in 2016. However, for other types of cancers, oncolytic viruses as effective cancer therapeutics are still under development. Intravenous injectable safe oncolytic viruses using virotherapeutics offer an ideal approach for cancer treatment, considering the challenges associated with direct injection of oncolytic viruses into tumors, especially in locations with numerous drug delivery challenges.^7^ Consequently, intravenous injectable oncolytic viruses are the preferred candidate for the treatment of metastatic cancer.^8^

Newcastle disease virus (NDV), also known as avian paramyxovirus type 1, belongs to the family *Paramyxoviridae* and has been explored for human cancer treatment because of its ability to infect and effectively propagate within various types of cancer cells, leading to cancer cell death.^9^ As a cancer virotherapeutic, NDV offers several potential benefits.^10^ Adenovirus and adeno‐associated virus (AVV) are DNA viruses widely used as virus treatment agents, however, NDV is RNA viruses, which do not have to be concerned about penetrating the patient's genes and is safe because it acts in the cytoplasm.^11^ The previous Phase I and Phase II clinical trials of NDV have shown its relative safety in humans, with mild side effects and low immune responses against NDV, even when administered intravenously.^12^, ^13^, ^14^ However, NDV infection has a mechanism of attacking cancer cells through an immune response through the activation of dendritic cells, cytokines, IL‐2, etc., so the possibility of a cytokine storm remains. Research is underway to find an appropriate treatment method that takes into the virus titer duration of administration and the patient's condition. NDV has been found to exert a broad spectrum of oncolytic effects on various types of cancer cells.^15^, ^16^, ^17^ Several studies have explored the relationship between NDV virulence and cancer cell‐killing mechanisms.^18^ The intrinsic oncolytic potential of NDV relies on its ability to selectively replicate in tumor cells and induce their death. This selectivity is linked to differences in activation of the type I interferon (IFN) response between tumor and normal cells. In normal cells, RNA virus clearance is initiated immediately after interferon activation, whereas this response is impaired in cancer cells, enabling NDV to target and destroy the tumor cells effectively.^19^ The mechanisms underlying tumor‐selective NDV replication and molecular‐level genomic RNA transcription and translation have been intensively investigated. NDV infection initiates HN protein binding to sialic acid on glycoproteins of the cell surface, but how NDV selectively infects cancer cells is unclear. Both lytic and nonlytic NDV strains show oncolytic properties. Normally, lentogenic strains are classified as nonlytic strains, while mesogenic and velogenic strains are classified as lytic strains. Nonlytic strains of NDV induce cell death by triggering the intrinsic and extrinsic apoptotic signaling pathways that cause cancer cells to begin their apoptosis. Lytic strains of NDV can infect and kill cancer cells as well as release newly formed progeny viruses.^20^, ^21^ The oncolytic effects and mechanisms of several low‐virulence NDV strains have been studied.^22^, ^23^, ^24^ The cancer cell‐killing mechanisms of non‐lytic NDV involve both intrinsic and extrinsic apoptotic pathways and stimulation of immune responses against NDV‐infected cancer cells. NDV infection induces cancer cell apoptosis by stimulating intrinsic and extrinsic signaling pathways. NDV infection leads to increased NF‐κB production, which in turn enhances the production of tumor necrosis factor‐related apoptosis‐inducing ligand (TRAIL). Soluble TRAIL forms trimers with a TNF homologous domain that binds to TRAIL death receptors (DR4 and DR5), further contributing to the apoptotic process.^25^ TRAIL and the TRAIL receptor complex play a vital role in activating FADD‐mediated caspase 8, initiating sequential caspase signal transduction, ultimately leading to cancer cell apoptosis.^26^ NDV infection initiates an intrinsic pathway to viral protein synthesis in cancer cells. Upon internalization of the NDV genomic RNA into the cancer cell cytoplasm, the NDV M protein interacts with the Bax protein, forming M‐Bax oligomers that bind to the mitochondrial membrane, leading to the release of cytochrome‐c. The released cytochrome‐c then forms an apoptosome, inducing caspase‐mediated apoptosis signal transduction in cancer cells.^27^

Since the identification of TRAIL, numerous studies have reported the functions of TRAIL and verified its ability to induce apoptosis in various cancer cells.^28^, ^29^ TRAIL is utilized for cancer therapeutics as either a recombinant TRAIL protein or by introducing TRAIL gene in cancer cells, aiming to induce apoptosis in cancer cells.^30^, ^31^ Soluble TRAIL molecules assemble into trimer complexes that bind to the TRAIL receptors present on the cell surface. Four different types of TRAIL receptors are expressed on the cell surface. Among them, TRAIL receptors DR4 (TRAIL‐R1) and DR5 (TRAIL‐R2) act as inducers of cancer cell apoptosis. TRAIL‐R3 (DcR1) is a no‐signaling receptor that lacks an intracellular domain, whereas TRAIL‐R4 (DcR2) has a truncated death domain and cannot initiate apoptosis.^32^, ^33^ In preclinical and clinical studies, cancer treatments often involve the application of recombinant TRAIL or specific monoclonal antibodies targeting TRAIL‐R1 and TRAIL‐R2 to cancer cells effectively. However, some current studies have revealed that certain cancer cells are resistant to TRAIL‐induced apoptosis. For example, HT‐29 is considered a TRAIL‐resistant CRC cell line owing to its lower expression of TRAIL‐R2, compared to other CRC cell lines, such as HCT116 (which is TRAIL‐sensitive). To overcome this resistance, researchers are exploring the use of various approaches, including combining chemicals or natural products with recombinant TRAIL or specific monoclonal antibodies targeting TRAIL receptors, although the synergistic cancer cell killing mechanism of these combinations remains unclear. Notably, Kan et al. demonstrated that NDV infection stimulates p53 expression in human cancer cells,^34^ which, in turn, transactivates TRAIL‐R2 gene expression through three intrinsic p53‐binding sequences.^35^ These findings suggest NDV infection can activate the expression of p53, which subsequently induces apoptosis‐related TRAIL‐R2 activation.

In this study, we used lentogenic strain‐based recombinant NDV containing human TRAIL gene to investigate the effect on the killing mechanism of TRAIL‐resistant CRC (HT‐29) cells, as well as TRAIL‐sensitive CRC (HCT116) cells. We primarily focused on determining the effect of NDV infection on DR expression in HT‐29 cell. Furthermore, we aimed to compare the effects of TRAIL gene expression on the intrinsic and extrinsic apoptotic signaling pathways in HT‐29 with those observed in HCT116.

## 2 MATERIALS AND METHODS

### 2.1 Cell lines and cell cultures

Human CRC cells (HT‐29 and HCT116) and African green monkey kidney cells (Vero) were purchased from the Korean Cell Line Bank (KCLB, Republic of Korea). Additionally, human larynx carcinoma cells (HEp‐2) were purchased from the American Type Culture Collection (ATCC, USA). HT‐29 and HCT116 cells were cultured in RPMI1640 medium supplemented with 10% (v/v) fetal bovine serum (FBS), 1% (v/v) penicillin–streptomycin solution (10 000 unit/mL of penicillin and 10 000 μg/mL of streptomycin, PS, Gibco, Billings, MT, USA). Vero cells were cultured in DMEM supplemented with 10% FBS and 1% PS. For HEp‐2 cells, MEM was used as the culture medium, supplemented with 10% FBS, and 1% PS.

### 2.2 cDNA construction of NDV containing TRAIL gene

The previously generated full‐length cDNA clone of recombinant NDV (rNDV), which contained the transgene cassette inserted between the NP and P genes of the non‐pathogenic NDV VG/GA strain), was used as the backbone to construct new cDNA.^22^, ^36^ The plasmid was linearized by restriction enzyme digestion to allow insertion of the extracellular form of the human TRAIL gene (amino acids 114–281). The TRAIL gene was amplified with NotI‐GE‐IG‐GS‐Kozak at the 3′‐end and FseI at the 5′‐end using the Phusion Flash High‐Fidelity PCR Master Mix (cat. No. F548S; Thermo Fisher Scientific, Inc., Waltham, MA, USA), resulting in a cloned insert between the NP and P genes, named p‐rNDV‐TRAIL. The PCR conditions were as follows: 1 cycle at 98°C for 30 s, followed by 30 cycles at 98°C for 10 s, 50°C for 30 s, and 72°C for 30 s.

### 2.3 Rescue of recombinant NDV‐TRAIL virus

HEp2 cells were seeded at density of 5 × 10^5.0^ cells/well in a 6‐well cell culture plate. After 1 day, the cells were infected with modified vaccinia virus (MVA/T7) at a multiplicity of infection (MOI) of 1.0 and incubated at 37°C with 5% CO_2_ for 2 h. Subsequently, the cells were washed twice with phosphate buffered saline (PBS, pH 7.4), followed by the addition of 2.0 mL of MEM (Gibco) containing 1% FBS (Sigma‐Aldrich, St. Louis, MO, USA) and 1% PS (Gibco). For transfection, a mixture of Lipofectamine 3000 (Invitrogen, Waltham, MA, USA), P3000 reagent (Invitrogen), p‐rNDV‐TRAIL (5 μg), and three helper plasmids (NP 2.5 μg, P 1.5 μg, and L 0.5 μg, respectively) per well was added to HEp‐2 cells and incubated at 37°C in a 5% CO_2_ incubator, according to the manufacturer's instructions. After 4 days of transfection, the supernatant was harvested. To propagate the virus, the allantoic cavity of 8‐ to 10‐day‐old embryonated specific pathogen‐free (SPF) eggs was inoculated with 300 μL of the harvested supernatant. After 4 days of inoculation, allantoic fluid was harvested and used for repeated inoculation into the allantoic cavity to eliminate the vaccinia virus. The supernatant was cultured and adapted to Vero cells.

### 2.4 Analysis of viral growth

Viral growth was detected in both HCT116 and HT‐29 cell lines. For the experiment, cells were seeded in a 6‐well plate and infected with a viral load of 0.1 MOI of rNDV or rNDV‐TRAIL viruses. The supernatants were harvested at 12, 24, 36, 48, 60, 72, 84, and 96 h postinfection. Subsequently, the viral titer was determined at 50% tissue culture infective dose per mL using Vero cells as the indicator cells.

### 2.5 Cell viability assay

Cell viability was assessed using a microculture tetrazolium (MTT) assay. Approximately 1 × 10^4.0^ HCT116 and HT‐29 cells were seeded in each well of 96‐well plates containing RPMI1640 supplemented with 10% FBS and 1% PS. Subsequently, the cells were infected with rNDV or rNDV‐TRAIL at different MOIs of 0.01, 0.1, 1, and 2.5 or with rNDV (10 MOI), rNDV‐TRAIL (10 MOI), rNDV (10 MOI) with TRAIL protein (100 ng/mL), and TRAIL protein (100 ng/mL), with each experiment performed in triplicate. After incubating for 24, 48, 72, and 96 h, 20 μL of MTT solution (CellTiter 96® AQueous One Solution Cell Proliferation Assay, Promega, Madison, WI, USA) was added to each well. Following an additional 1 h incubation, the cell death was measured at 490 nm (OD490) using an iMark Microplate Reader (cat. No. 1681130EDU, Bio‐Rad, Hercules, CA, USA). Thereafter, the relative death rate (%) of the control group was calculated to determine the impact of the treatments in cell viability.

### 2.6 Sample preparation

One day before virus inoculation, HCT116 or HT‐29 cells were seeded in a 75T flask at a density of 5 × 10^6.0^ cells/flask. The cells were then infected with rNDV or rNDV‐TRAIL virus at an MOI of 1.0. After 12, 24, and 36 h, the proteins were extracted from the cells using 3 cycles of freezing and thawing performed at −80 and 4°C, respectively. The resulting cell lysates were used for immunoblot analysis to examine the expression of specific proteins.

### 2.7 Immunoblotting

The extracted proteins were mixed with sample buffer (250 mM Tris–HCl pH 6.8, 5% 2‐mercaptoethanol, 10% SDS, 0.5% bromophenol blue, 50% glycerol). The mixture was denatured at 100°C for 3 min and then subjected to sodium dodecylsulfate‐poly acrylamide gel electrophoresis (SDS‐PAGE). Subsequently, the separated proteins were transferred to a polyvinylidene difluoride (PVDF) membrane using a Power Blotter (cat. no. PB0012, Thermo Fisher Scientific, Inc., Waltham, MA, USA) at 25 A for 15 min. The PVDF membranes were blocked by 1% BSA for 1 h and then probed with the following antibodies: anti‐GAPDH (sc‐32233), anti‐P53 (sc‐126), anti‐DR5 (sc‐166624) (all from Santa Cruz Biotechnology, Santa Cruz, CA, USA; used at a dilution of 1:1000); anti‐Bcl‐2 (ab182858), anti‐Bax (ab270742), anti‐Cytochrome C (ab133504), anti‐Caspase‐9 (ab202068), anti‐Caspase8 (ab220171), anti‐Caspase3 (ab32351) (all from Abcam, Cambridge, UK; used at 1:1000); and anti‐TRAIL (55B709.3) (Novus Biologicals, Briarwood, USA; used at 1:1000). After incubation with the primary antibodies, the membranes were treated with enhanced chemiluminescence (ECL) substrates A and B in a 1:1 ratio. The resulting images were captured using the ChemiDoc™ MP Imaging System (cat. no. 17001402 Bio‐Rad, Hercules, CA, USA), and the band densities were semiquantitatively analyzed using Image J V.1.8.0.

### 2.8 Animals

All animal experiments conducted in this study were approved by the Animal Care and Use Committees of Libentech Co., Ltd (LBT‐IACUC‐AE‐2020‐01). Five‐week‐old female BALB/c nu‐/nu‐ mice were purchased from Orient Bio (Seoul, Republic of Korea). Throughout the study, the mice were housed in controlled conditions, with an ambient temperature of 22 ± 1°C and a light/dark cycle of 12 h. They were provided with free access to sterilized food and water and monitored regularly for signs of distress or adverse effects. A body‐weight reduction of 20% or more was considered a human endpoint, at which the mice were sacrificed to minimize potential suffering and ensure animal welfare during the experimental period.

### 2.9 Animal studies

For the xenograft assay, suspended HCT116 (1 × 10^6.0^ cells/dose) or HT‐29 cells (5 × 10^6.0^ cells/dose) in sterile PBS were mixed with 100 μL matrigel (cat. No. 354230, Corning, NY, USA) and injected into the left flanks of nude mice. Tumor formation was monitored by caliper measurements every 2 days. Tumor volume was calculated using the formula 1/2 × (smallest diameter)^2^ × (largest diameter). When the tumor volume reached an average of 100–200 mm^3^, the mice were randomly divided into the following groups and intravenously inoculated accordingly at 1, 3, 5, 7, and 9 days: Control group (100 μL sterile PBS), rNDV group (1 × 10^7.0^ TCID_50_/dose rNDV viruses), rNDV‐TRAIL group (1 × 10^7.0^ TCID_50_/dose rNDV‐TRAIL viruses). The tumor volumes were monitored every 2 days using a caliper. At the end of the experiments, the mice were sacrificed, and the tumors were collected for immunoblotting and histological analyses.

### 2.10 Immunohistochemical and TUNEL assay

The tumor tissues were fixed using a 10% (w/v) formalin solution and then processed for paraffin embedding. Tumor blocks were dehydrated using ethanol and xylene, and 4‐μm‐thick sections were prepared. For immunohistochemical (IHC) staining, specific tissue antigens were localized. The sections were incubated overnight at 4°C with primary antibodies against TRAIL (1:200, ab231265, Abcam, Cambridge, UK) and NDV HN (1:1600, BS‐4529R, Bioss, USA). The color was visualized using 3,3′‐diaminobenzidine (DAB). For the TUNEL assay (ab206386, Abcam, Cambridge, UK), the sections were processed according to the manufacturer's instructions to detect DNA fragmentation indicative of apoptosis. All images were analyzed using Image J V.1.8.0 to quantify the results.

### 2.11 Statistical analysis

Statistical analysis was performed using Prism 8 software (GraphPad Software Inc., La Jolla, CA, USA). The data are presented as the mean ± standard error of the mean (SEM). Statistical comparisons were evaluated using Student's *t*‐test. *p*‐values <0.05 were considered to indicate a statistically significant difference between groups.

## 3 RESULTS

### 3.1 Generation of recombinant NDV expressing human TRAIL gene

**FIGURE 1:**
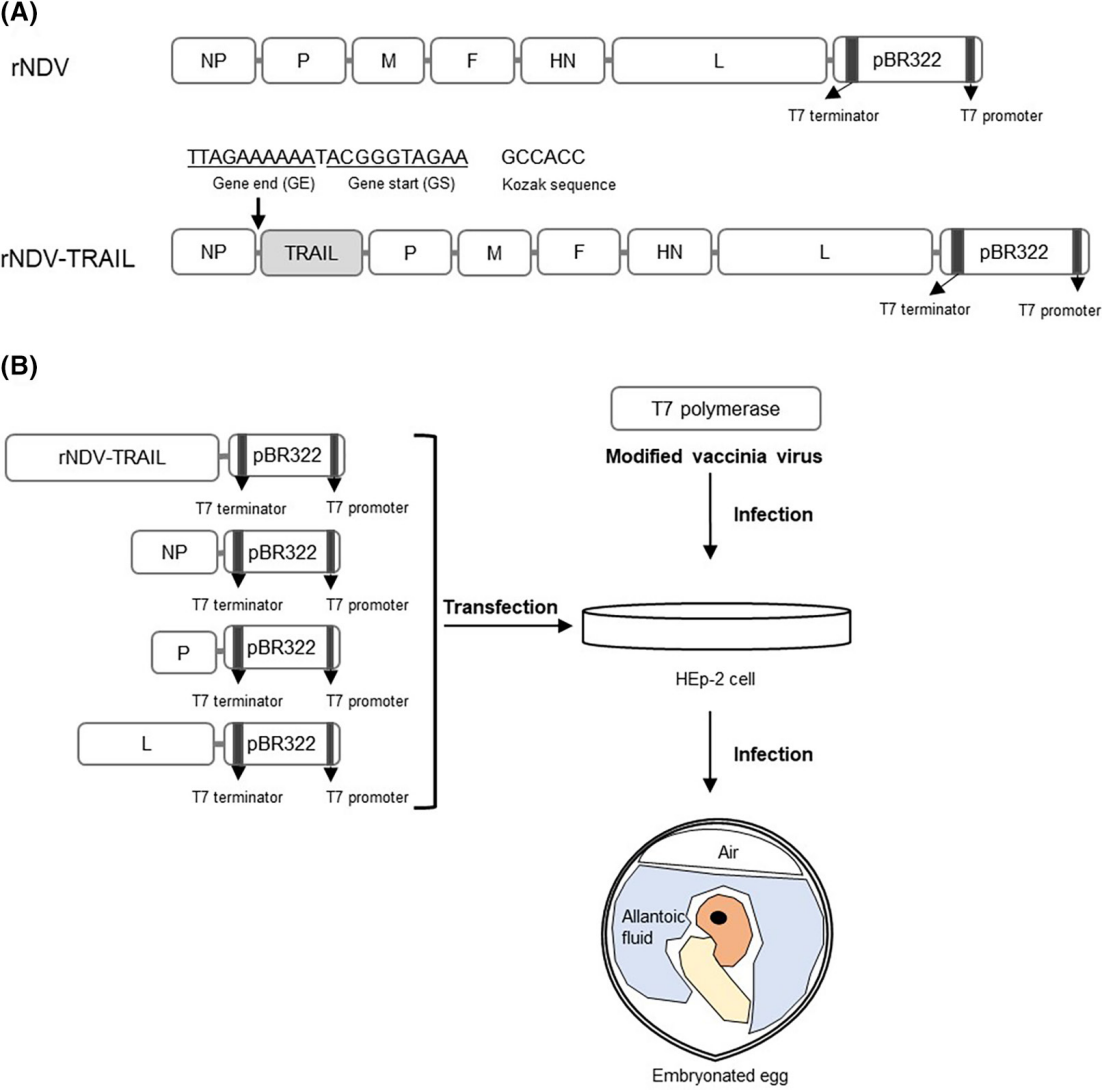
Construction of the recombinant NDV and NDV‐TRAIL viruses. (A) Schematic representation of NDV VG/GA strain‐based recombinant NDV (rNDV) and rNDV containing TRAIL gene (rNDV‐TRAIL) vector. The previously generated cDNA, rNDV‐PTEN^20^, ^33^ was used as backbones to the product vector containing TRAIL gene between NP and P gene. (B) Recovery of an infectious NDV from cDNA. The helper plasmids (p‐NP, p‐P, and, p‐L) were co‐transfected with p‐rNDV‐TRAIL to HEp‐2 cells. The supernatant was inoculated into SPF eggs. The allantoic fluid was harvested and adapted to Vero cells.

Recombinant NDV (rNDV) and rNDV containing the human TRAIL gene (rNDV‐TRAIL) viruses were generated according to the process illustrated in Figure 1A. After 4 days of co‐transfection of the p‐rNDV‐TRAIL and helper plasmids (p‐NP, p‐P, p‐L) into HEp‐2 cells, the rNDV‐TRAIL virus was rescued as described in the section 2 (Figure 1B). The nucleotide sequence of the viruses was confirmed through sequence analysis of the RT‐PCR products of the genomes (data not shown).

### 3.2 Enhanced oncolytic activity of rNDV‐TRAIL compared to rNDV in TRAIL‐resistant CRC cells

**FIGURE 2:**
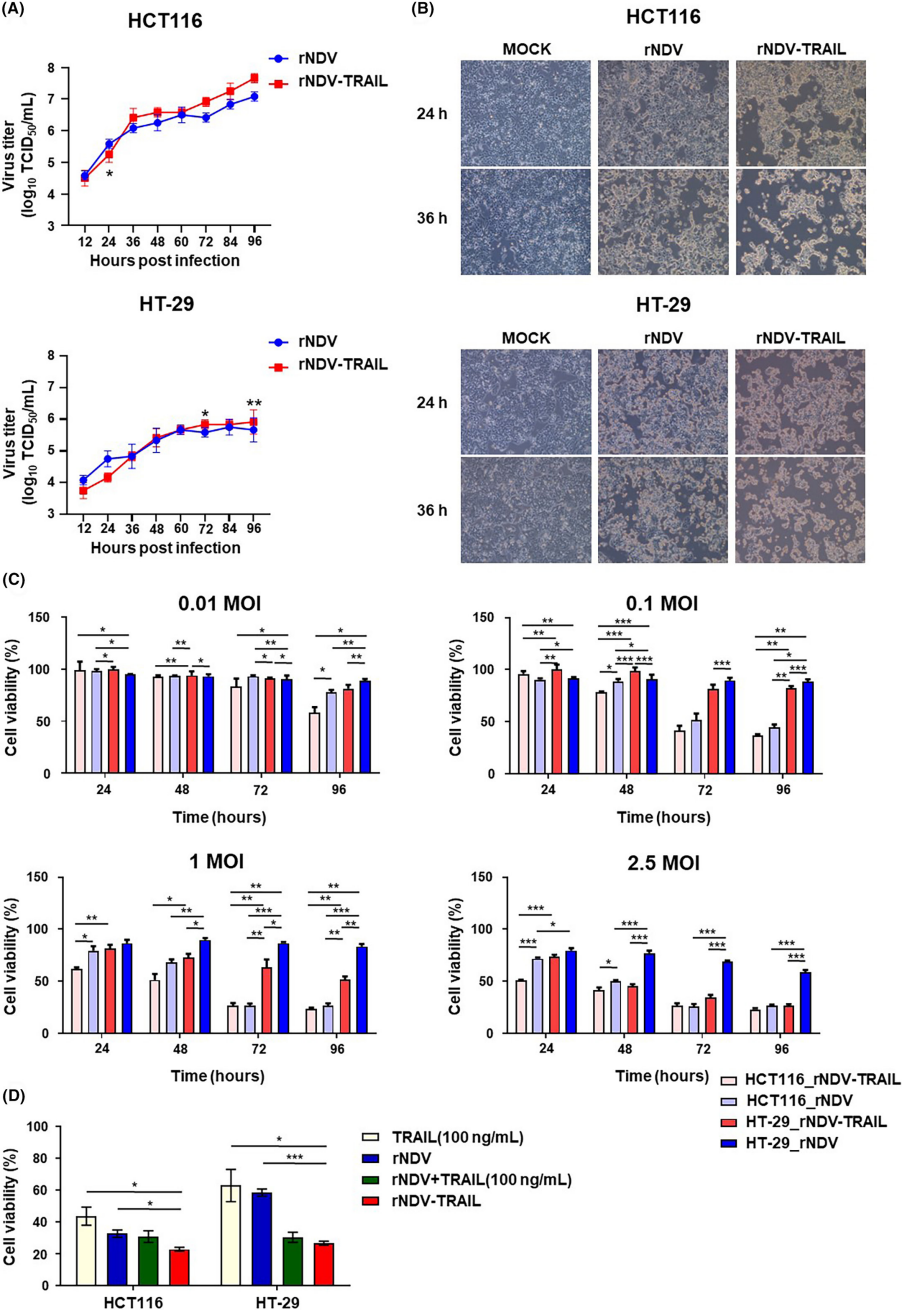
The susceptibility of colorectal cancer cells to rNDV or rNDV‐TRAIL infections. (A) Growth kinetics of rNDV and rNDV‐TRAIL viruses in HCT116 and HT‐29 cell lines. The cells were respectively infected with rNDV and rNDV‐TRAIL viruses at an MOI of 0.1. The supernatant was collected at 12, 24, 36, 48, 60, 74, 84, and 96 h postinfection for TCID_50_ titration on Vero cells in triplicates. The graph was expressed in mean log_10_TCID_50_/mL with a deviation and error bars represent the standard deviation of the mean (n = 3). (B) CPE of rNDV and rNDV‐TRAIL on HCT116 and HT‐29 cells at 24 and 36 h postinfection. Images were obtained under the microscope. (C) MTT assays of rNDV and rNDV‐TRAIL on HCT116 and HT‐29 cell lines. Before 1 day, the cells were cultured in 96‐well tissue culture plates and infected with several titers (0.01, 0.1, 1, and 2.5 MOI) of rNDV or rNDV‐TRAIL viruses, as indicated. For negative control, the cells were grown in a culture medium. After 24, 48, 72, or 96 h postinfection, MTT assays were performed Cell Proliferation Assay kit. (D) MTT assays of rNDV, rNDV‐TRAIL, rNDV with TRAIL protein (100 ng/mL), and TRAIL protein (100 ng/mL) on HCT116 and HT‐29 cell lines. Before 1 day, the cells were cultured in 96‐well tissue culture plates and infected with 10 MOI of rNDV, rNDV‐TRAIL viruses, rNDV with TRAIL protein (100 ng/mL), or TRAIL protein (100 ng/mL). For negative control, the cells were grown in a culture medium. After 96 h postinfection, MTT assays were performed Cell Proliferation Assay kit. These data (C, D) are presented as means ± the standard deviation of four independent experiments. *p < 0.05, **p < 0.01, ***p < 0.001.

To assess the virus growth curve, both rNDV and rNDV‐TRAIL viruses were infected in Vero (Figure S1), HCT116, and HT‐29 cells, and the cell culture medium was collected at 0, 12, 24, 36, 48, 60, 72, 84, and 96 h postinfection (h.p.i.). The viral titer was determined using the TCID_50_ assay in Vero cells. As shown in Figure 2A, the viral titer gradually increased in a time‐dependent manner. In HCT116 cells infected with rNDV, the viral titer peaked at 96 h.p.i. to a value of 10^7.08^ TCID_50_/mL, while in HCT116 cells infected with rNDV‐TRAIL, the viral titer peaked to 10^7.67^ TCID_50_/mL, also at 96 h.p.i. Similarly, in HT‐29 cells, the viral titer peaked at 10^5.75^ TCID_50_/mL at 84 h.p.i. when infected with rNDV and to 10^5.91^ TCID_50_/mL at 96 h.p.i. when infected with rNDV‐TRAIL. In Vero cells, both rNDV and rNDV‐TRAIL infection led to an increase in viral titer over time, which peaked at 48 h.p.i. for both viruses (10^6.62^ TCID_50_/mL for rNDV and 10^6.80^ TCID_50_/mL for rNDV‐TRAIL), followed by reaching a plateau at 72–96 h.p.i. These results suggest that the insertion of the TRAIL gene did not affect viral growth in the tested cells.

To compare the oncolytic effect of rNDV and rNDV‐TRAIL infection on TRAIL‐resistant CRC cells, HCT116 and HT‐29 cells were separately infected with the viruses at an MOI of the cytopathic effect (CPE) was monitored and observed under a microscope (Figure 2B). Over the course of infection, the cells infected with rNDV and rNDV‐TRAIL displayed more irregular cellular morphology. Notably, the HCT116 and HT‐29 cells infected with rNDV‐TRAIL virus showed the most pronounced irregular cellular morphology at 36 h.p.i.

To assess the oncolytic effect and induction of apoptosis, an MTT assay was performed in HCT116 and HT‐29 cell lines (Figure 2C). The results showed that the apoptotic effect was directly proportional to the virus MOI. Nevertheless, even with low MOI virus inoculation, the apoptotic effect was higher in HCT116 cells than in HT‐29 cells at 72 and 96 h.p.i. At 96 h.p.i., the percentages of apoptosis at 2.5 MOI for rNDV and rNDV‐TRAIL were 26.87% and 22.94% in HCT116 cells and 58.56% and 26.8% (*p* < 0.001) in HT‐29 cells, respectively. Interestingly, the oncolytic effect of rNDV‐TRAIL virus in HT‐29 cells significantly increased by 2.18‐fold compared to rNDV infection, indicating that rNDV‐TRAIL promotes TRAIL‐induced apoptosis in TRAIL‐resistant HT‐29 cells. These results reveal that rNDV‐TRAIL exhibits a significantly enhanced oncolytic effect against HCT116 and HT‐29 compared to rNDV alone.

In addition, to evaluate TRAIL‐induced apoptosis, HCT116, and HT‐29 cells were treated with TRAIL protein (100 ng/mL), rNDV, rNDV‐TRAIL, and rNDV with TRAIL protein (100 ng/mL) at an MOI of 10 for 96 h, and their viability was assessed using the MTT assay (Figure 2D). The percentages of growth inhibition were 43.7%, 32.7%, 30.9%, and, 22.9% in HCT116 cells and 62.9%, 58.6%, 30.4%, and, 26.8% in HT‐29 cells infected with TRAIL protein (100 ng/mL), rNDV (10 MOI), rNDV (10 MOI) with TRAIL protein (100 ng/mL), and rNDV‐TRAIL (10 MOI), respectively. Notably, the TRAIL‐resistant HT‐29 cells infected with rNDV (10 MOI) with TRAIL protein (100 ng/mL) and rNDV‐TRAIL virus‐induced apoptosis more effectively than rNDV. These findings align with the results of the MTT assay. Furthermore, the data confirmed that rNDV‐TRAIL virus infection was more effective in inducing apoptosis than rNDV with TRAIL protein (100 ng/mL) in both HCT116 and HT‐29 cells. The rNDV‐TRAIL virus infection of the HCT116 and HT‐29 cells increased TRAIL reactivity, and the TRAIL protein generated by the rNDV‐TRAIL virus induced more pronounced apoptosis.

This study compared the oncolytic effects of the rNDV‐TRAIL virus on TRAIL‐resistant HT‐29 cells with TRAIL‐resistant HCT‐116 cells as a control. As shown in Figure 2A, both rNDV and rNDV TRAIL exhibited more active virus multiplication in HCT 116 cells compared to HCT‐29 cells. This finding is consistent with our observation that the apoptosis effect in cancer cells infected at an MOI of <1 is proportional to the virus multiplication level. Moreover, this study showed that rNDV‐TRAIL virus at various MOI levels had a more potent oncolytic effect on both HCT 116 and HCT 29 cells than the rNDV virus alone (Figure 2C). Based on these results, it was speculated that the expression of the TRAIL protein was significantly more active in rNDV‐TRAIL compared to rNDV, leading to enhanced apoptosis effect. Furthermore, in Figure 2D, the MTT assay result confirmed that the TRAIL protein induced by rNDV‐TRAIL exerted more pronounced cancer cell apoptosis effectiveness compared to rNDV and recombinant TRAIL protein (100 ng/mL).

### 3.3 rNDV infection upregulates TRAIL and DR5 expression in TRAIL‐resistant CRC cells

**FIGURE 3:**
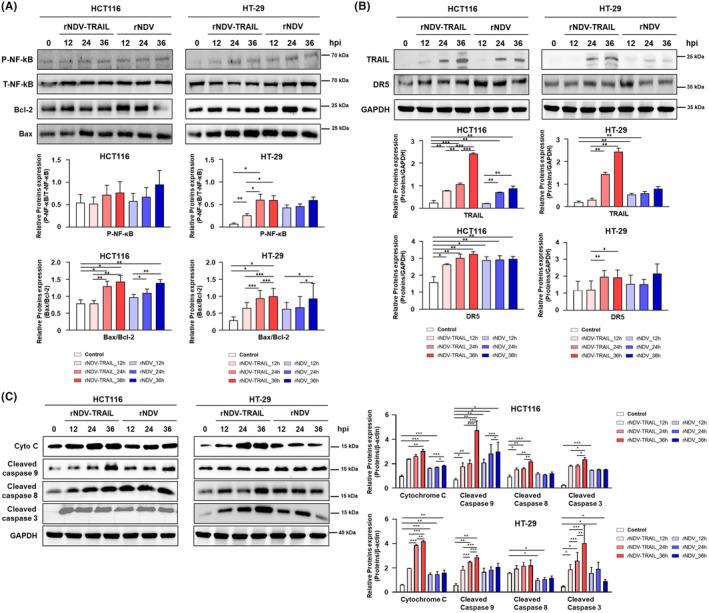
rNDV‐TRAIL virus upregulates TRAIL‐sensitivity and effectively induces apoptosis pathway. (A) Upregulation by rNDV or rNDV‐TRAIL infection on expression and activation of NF‐κB‐p, TRAIL, P53, and DR5. HCT116 and HT‐29 cells were infected with rNDV or rNDV‐TRAIL, and harvested at 12, 24, and 36 h postinfection. The rNDV or rNDV‐TRAIL induced apoptosis was analyzed with western blot analysis using anti‐NF‐κB, anti‐NF‐κB‐p, anti‐TRAIL, anti‐P53, and, anti‐DR5. (B) Activation of intrinsic and extrinsic apoptosis by rNDV and rNDV‐TRAIL infection. The rNDV or rNDV‐TRAIL induced apoptosis was analyzed with western blot analysis using anti‐bcl‐2, anti‐bax, anti‐cytochrome C, anti‐caspase 9, anti‐caspase 8, and, anti‐caspase 3. GAPDH was loaded as control. The intensities of bands were analyzed by densitometry using ImageJ software, normalized to GAPDH, and presented as means ± the standard deviation of four independent experiments. *p < 0.05, **p < 0.01, ***p < 0.001 vs control.

A previous study showed that rNDV infection stimulates the transcription of TNF‐α and TRAIL via NF‐κB.^37^ To investigate the mechanism by which rNDV acts in TRAIL‐resistant cell lines, the levels of total NF‐κB (T‐NF‐κB), phosphorylated NF‐κB (P‐NF‐κB), Bcl‐2, and Bax expression were analyzed by western blotting in both HCT116 and HT‐29 cells (Figure 3A). To detect the cellular response to viral infection, we investigated NF‐κB and P‐NF‐κB levels. The increased P‐NF‐κB levels were detected from 12 to 36 h.p.i. in rNDV‐infected HCT116 cells at 12 h.p.i. During virus infection, the downregulation of Bcl‐2 (26 kDa), accompanied by an increase in Bax (21 kDa), which led to an increase in Bax/Bcl‐2 ratio, was detected from 12 to 36 h.p.i.

In both HCT116 and HT‐29 cells, the levels of DR5 expression were analyzed by western blot analysis (Figure 3B). At 24 and 36 h.p.i., the expression of TRAIL by rNDV‐TRAIL was higher than that by rNDV. These results showed that rNDV‐TRAIL infection significantly increased TRAIL expression in a time‐dependent manner. Furthermore, we tested whether the expression levels of DR5 were increased in HCT116 and HT‐29 cells. Collectively, these results verify that NDV sensitizes CRC cells to TRAIL by inducing the expression levels of DR5.

### 3.4 rNDV expressing TRAIL enhances the induction of both intrinsic and extrinsic apoptosis in TRAIL‐resistant CRC cells

Previous studies have reported that NDV induces both extrinsic and intrinsic apoptosis.^38^ To compare the characteristics of apoptosis induced by rNDV and rNDV‐TRAIL viruses, we tested caspase activation in virus‐infected HCT116 and HT‐29 cells using western blot analysis. As shown in Figure 3C, the cleaved caspase eight bands were detected in all virus‐infected cells from 12 h.p.i., indicating activation of the extrinsic apoptosis pathway. Additionally, the cleavage of caspase 9 by cytochrome C promotes intrinsic apoptosis. The results showed that from 12 to 36 h.p.i., the cytochrome C (14 kDa) levels increased, along with an increase in the levels of cleaved caspase 9 (17 kDa), suggesting activation of the intrinsic apoptosis pathway. Caspase 3, which is implicated in both intrinsic and extrinsic signaling pathways, was also observed to be cleaved in virus‐infected cells. Finally, a cleaved caspase 3 fragment (17 kDa) was detected from 12 h.p.i. in HCT116 and HT‐29 cells, confirming that apoptosis was induced through both extrinsic and intrinsic pathways in both cell lines. Interestingly, the cleaved caspase 3 levels in HT‐29 cells infected with the rNDV‐TRAIL virus were more pronounced than in HCT116 cells. In TRAIL‐resistant HT‐29 cells, rNDV‐TRAIL infection significantly activated caspases‐8, 9, and 3 and the release of cytochrome C, compared to rNDV. These results suggest that the rNDV‐TRAIL virus enhances TRAIL sensitivity and effectively activates apoptosis in TRAIL‐resistant HT‐29 cells, compared to TRAIL‐nonresistant HCT116 cells.

### 3.5 rNDV‐TRAIL shows a stronger oncolytic effect than rNDV in TRAIL‐resistant CRC cell (HT‐29) xenograft

**FIGURE 4:**
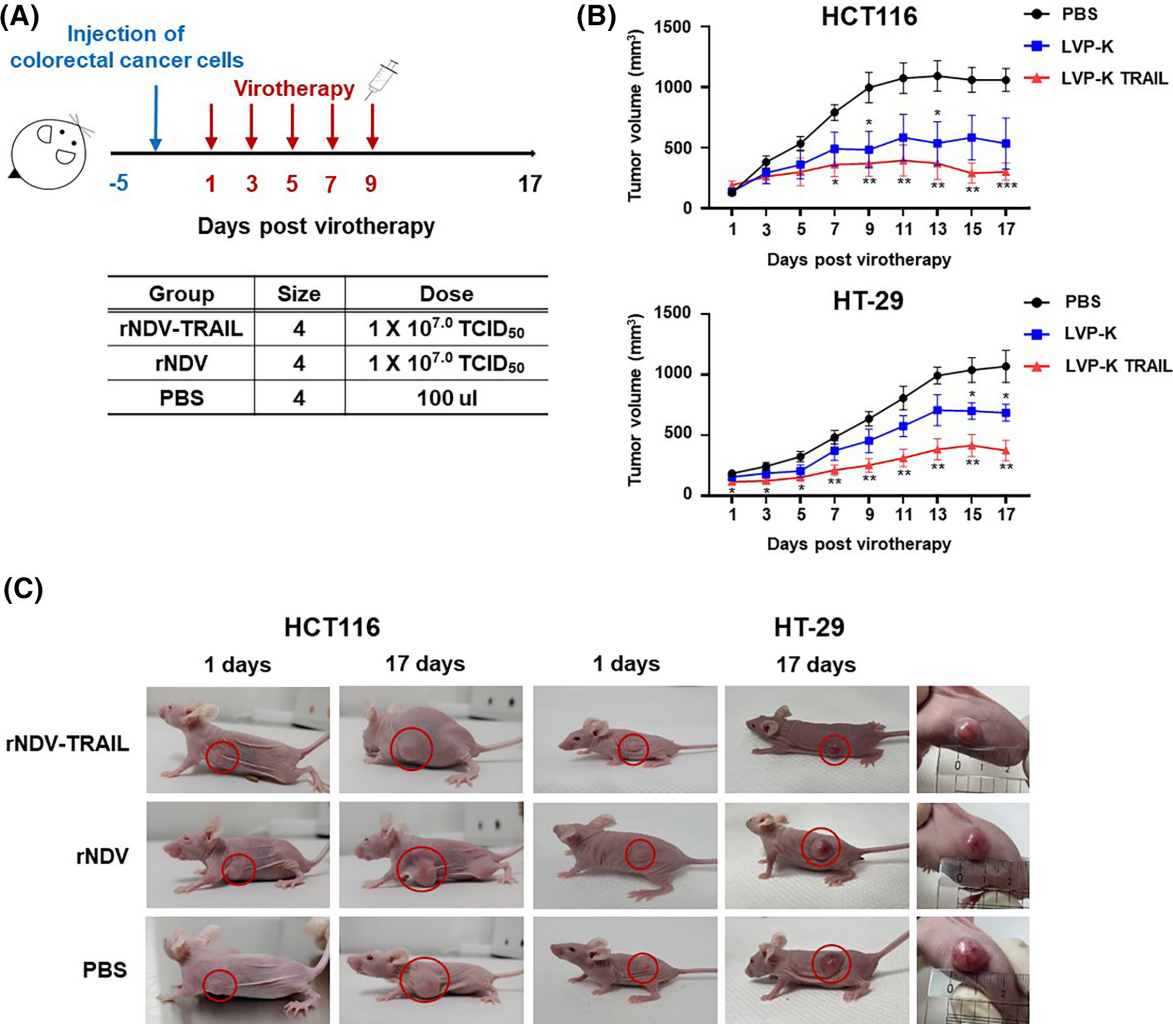
rNDV‐TRAIL viruses show enhanced oncolytic effect in vivo. (A) Mice were subcutaneously injected with HCT116 or HT‐29 cells. Five days postimplantation, the tumors were inoculated with five intravenous injections of PBS, rNDV, and rNDV‐TRAIL at 10^7.0^ TCID_50_ every 2 days. (B) Tumor volumes were repeatedly measured until Day 17 every 2 days, plotted, and presented as means ± the standard deviation. *p < 0.05, **p < 0.01, ***p < 0.001 versus PBS injected mice. (C) The HCT116 or HT‐29 xenograft mice inoculated with rNDV or rNDV‐TRAIL showed macroscopic appearance after 1 and 17 days postinjection.

These results confirmed that rNDV‐TRAIL infection enhanced the expression of p53 and DR5 and effectively induced apoptosis in vitro. To further investigate the differences caused by rNDV and rNDV‐TRAIL virus infection in vivo, we constructed a xenograft model using both TRAIL‐resistant (HT‐29) and nonresistant (HCT‐116) CRC cells. HCT116 and HT‐29 cells were implanted into the left flank of BALB/c nu‐/nu‐ mice (Figure 4A). When the tumor volume reached 100–200 mm^3^, mice were injected with a 100 μL suspension containing 1 × 10^7.0^ TCID_50_ of either rNDV or rNDV‐TRAIL viruses every 2 days for a total of five injections. Negative and positive controls were PBS and rNDV, respectively. Figures 4B,C present the tumor volume and images of the mice on 1‐ and 17‐days postinjection. These results showed the smallest tumor masses were observed in the rNDV‐TRAIL group (301.0 and 373.8 mm^3^), compared to the rNDV (533.7 and 685.6 mm^3^) or PBS (1059.3 and 1069.6 mm^3^) groups in both HCT116 and HT‐29 xenograft mice at 17 days postinjection. The growth in tumor volume was significantly suppressed by both rNDV and rNDV‐TRAIL.

**FIGURE 5:**
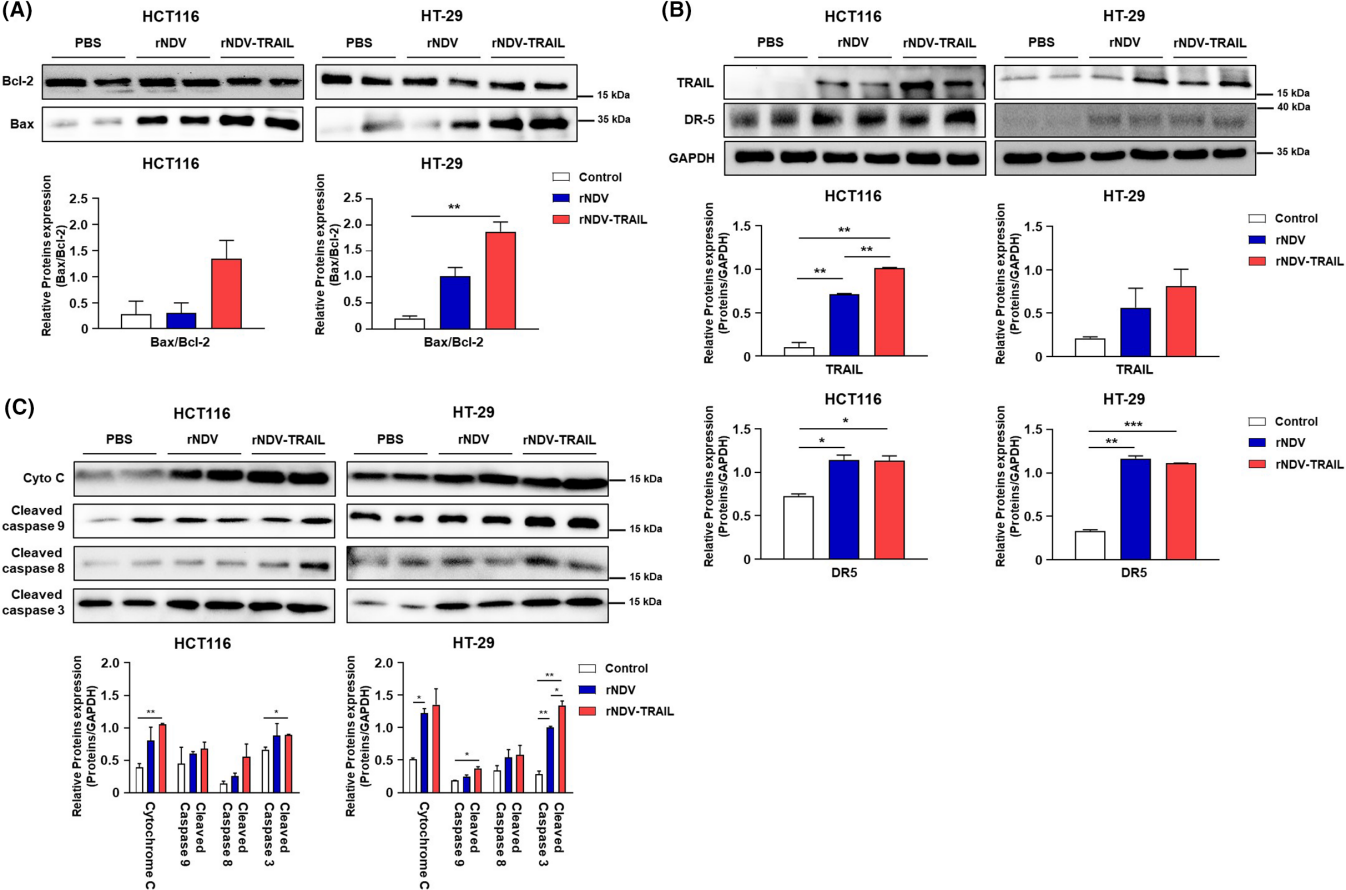
rNDV‐TRAIL viruses upregulate TRAIL‐sensitivity and induce more apoptotic signals in vivo. The tumors were collected and prepared for western blot analysis after 17 days post‐injection. (A) The expression and activation of NF‐κB‐p, TRAIL, P53, and DR5 were detected by western blot analysis in HCT116 and HT‐29 tumor tissue. (B) Apoptosis markers were identified by Western blot analysis in HCT116 and HT‐29 tumor tissue. GAPDH was loaded as internal control. The protein levels were analyzed by densitometry using ImageJ software, normalized to GAPDH, and presented as means ± the standard deviation of four independent experiments. *p < 0.05, **p < 0.01, ***p < 0.001 versus PBS injected mice.

To evaluate the effects of rNDV on the activation of apoptosis in vivo, we performed western blot analysis (Figure 5A–C). The results showed that the expression levels of TRAIL and DR5 significantly increased, and the activation levels of both extrinsic and intrinsic signaling pathways were enhanced in the rNDV and rNDV‐TRAIL groups compared to the PBS group in the HCT116 and HT‐29 tumor tissues. Moreover, in the rNDV‐TRAIL group, the activation levels of Bax/Bcl‐2 ratio, Cytochrome C, and caspase 9 of the intrinsic signaling pathway were increased compared to the rNDV group in both HCT116 and HT‐29 tumor tissues. The levels of cleaved caspases‐8 and 3 in rNDV‐TRAIL‐infected tumor tissues were higher than those in rNDV‐infected tumor tissues. In addition, the bands corresponding to Bax/Bcl‐2 ratio, cytochrome C, and caspase 3 were stronger in rNDV‐TRAIL‐infected HT‐29 tissues were detected than in rNDV‐TRAIL‐infected HCT116 tissues. These findings suggest that the TRAIL protein expressed by the rNDV‐TRAIL virus overcame TRAIL resistance and enhanced the oncolytic effect on TRAIL‐resistant HT‐29 cells.

**FIGURE 6:**
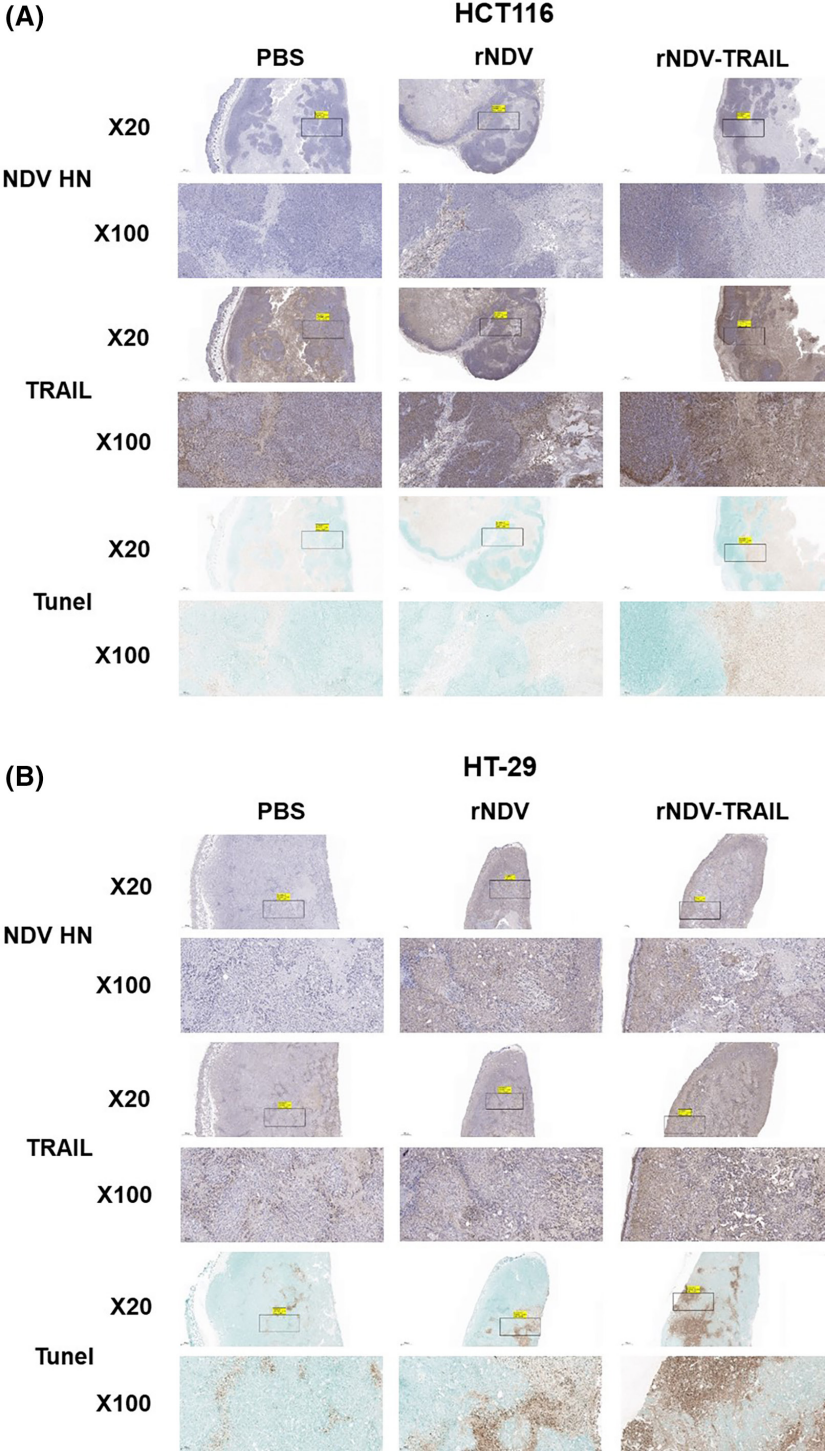
Histology and immunohistochemistry of (A) HCT116 and (B) HT‐29 xenograft tumors. The representative images of immunohistochemistry for NDV HN and TRAIL are shown in brown. TUNEL assay (terminal deosynucleotidy transferase‐mediated dUTP nick and labeling assay) was performed to detect the apoptosis of HCT116 or HT‐29 tumor cells and the results were detected in brown. The larger the brown area, the more serious the apoptosis of tumor cells was. Representative fields of view were shown at ×20 and ×100 magnification. Bar = 100 μm.

The expression levels of TRAIL and the HN protein of NDV were detected in tumor tissues using an IHC assay. In both HCT116 (Figure 6A) and HT‐29 (Figure 6B) xenografts mice, we observed a significant induction of TRAIL expression in the rNDV‐TRAIL group. Both rNDV and rNDV‐TRAIL groups showed the presence of HN proteins in the tumor tissues of mice, whereas the PBS group showed no expression of these proteins. Furthermore, we assessed the cytotoxic effect of the rNDV and rNDV‐TRAIL viruses on tumors using the TUNEL assay. Compared to the PBS group, both rNDV and rNDV‐TRAIL infections led to an increase in apoptotic cells in the tumors of both HCT116‐ and HT‐29‐implanted mice. Notably, rNDV‐TRAIL infection resulted in more apoptotic cells than rNDV infection in both types of tumors with a more pronounced effect observed in HT‐29‐implanted mice. The increased levels of HN and TRAIL proteins in the rNDV and rNDV‐TRAIL groups compared to the PBS group (*p* < 0.05) further support the inhibitory effect of tumor growth through apoptosis induction, as indicated by the histopathological analysis.

## 4 DISCUSSION

The oncolytic effect of NDV has been known since the 1950s, but significant advances in utilizing NDV for cancer therapy emerged with the introduction of reverse genetics technologies. Genetically engineered recombinant NDV (rNDV) has been explored for cancer therapy, utilizing both lytic and non‐lytic strains in human cells, as both strains efficiently replicate in human neoplastic cells. However, it is important to note that most non‐lytic strains known, which are avirulent to avian hosts, are unable to produce infectious progeny in human neoplastic cells.^39^ To enhance the oncolytic effect of rNDV, several researchers have utilized human tumor suppressor genes. Traditionally, these genes were inserted between the P (phosphoprotein gene) and M (matrix protein gene) based on the idea that this site is the most favorable for foreign gene insertion.^40^ However, recent studies have shown that the optimal insertion site may vary depending on the type of foreign gene being used and the characteristics of infected host cells.^41^ In this study, the TRAIL gene was inserted between NP (nucleocapsid protein gene) and P gene of the NDV genome, based on a previous study.^22^ The newly generated rNDV containing the TRAIL gene (rNDV‐TRAIL) exhibited similar growth kinetics to the rNDV without the TRAIL gene (Figure S1). Notably, TRAIL protein expression increased in HCT116 and HT‐29 cells infected with rNDV‐TRAIL compared to those infected with rNDV alone (Figure 3A). Although this study did not directly compare the expression levels of TRAIL according to the TRAIL gene insertion location in the NDV genome, the results support the successful application of the NP and P gene positions as effective sites for foreign gene insertion without causing severe difficulties in virus growth kinetics or TRAIL expression.

Upon infection, both rNDV or rNDV‐TRAIL stimulated NF‐κB phosphorylation in both HCT116 and HT‐29 cells, as depicted in Figure 3A. However, HT‐29 cells exhibited higher levels of NF‐ κB activation than HCT116 cells, relative to their respective uninoculated controls (Figure 3A). The activation of the NF‐κB pathway by NDV infection can lead to the upregulation of various genes involved in inflammation and immunity, including cytokines, chemokines, and adhesion molecules. Additionally, it can activate the expression of genes encoding anti‐apoptotic proteins, such as Bcl‐2, which can inhibit apoptosis and promote cell survival. In contrast, the tumor suppressor protein p53 is a known transcriptional target of the NF‐κB pathway. Activation of NF‐κB can lead to the upregulation of the MDM2 (mouse double minute 2) gene, which encodes a negative regulator of p53. However, in some cases, NF‐κB activation can also induce the expression of pro‐apoptotic proteins, such as PUMA and NOXA, which can activate p53 and promote apoptosis.^42^, ^43^ In both in vitro and in vivo experiments, rNDV or rNDV‐TRAIL infection activated BAX production and deactivated Bcl‐2 production, particularly evident in the BAX/Bcl‐2 ratio, which exhibited a more substantial increase in HT‐29 cells compared to HCT116 cells (Figure 3A and 5A). This result showed that NDV infection can induce apoptosis by upregulating the expression of pro‐apoptotic proteins, such as Bax, while downregulating the expression of anti‐apoptotic proteins, such as Bcl‐2. Consequently, this process activates caspases, which are proteases responsible for cleaving key cellular proteins, eventually leading to the disassembly of the cell.

Oncolytic NDV strains exert their anti‐cancer effects via both intrinsic and extrinsic caspase‐dependent cell death mechanisms.^44^, ^45^, ^46^ NDV infection induces the activation of TNF‐α, which, in turn, promotes the production of TRAIL protein. The released TRAIL protein binds to DRs present on the cell surface, initiating apoptosis through a series of caspase signaling pathways. Additionally, another intrinsic pathway involves the M protein of the infected NDV, which forms a complex with the BAX protein.^47^ This complex attaches to the mitochondrial membrane, leading to the release of cytochrome c, which then forms an apoptosome with Apaf‐1, subsequently activating caspase‐9 and initiating the downstream signaling cascade. Moreover, NDV infection activates the DR pathway of apoptosis by upregulating the expression of the TRAIL receptor. TRAIL is a protein that can induce apoptosis in cancer cells, but not in normal cells, making it an attractive target for cancer therapy. NDV infection upregulates the TRAIL receptor, sensitizing cancer cells to TRAIL‐induced apoptosis, thereby leading to their selective destruction. The extrinsic pathway of apoptosis is initiated when TRAIL binds to its DRs (DR4 or DR5) on the surface of cancer cells, triggering the formation of a death‐inducing signaling complex (DISC) that activates caspase‐8, which, in turn, activates downstream effector caspases (such as caspase‐3), leading to apoptosis. In addition to the extrinsic pathway, NDV infection can activate the intrinsic pathway of apoptosis by inducing the release of cytochrome c from mitochondria. The released cytochrome c activates caspase‐9 and downstream effector caspases, further driving the apoptotic process. In summary, NDV infection can activate both the intrinsic and extrinsic pathways of apoptosis, selectively inducing the destruction of cancer cells while sparing normal cells.

Several studies have shown the promising anti‐tumor activity of recombinant human TRAIL both in vitro and in vivo. However, in TRAIL‐resistant CRC cells, the effectiveness of recombinant TRAIL protein in killing cancer cells is limited, and clinical trials using recombinant TRAIL or TRAIL‐based combinations with antitumor drugs have not yielded the expected outcomes.^48^, ^49^, ^50^, ^51^ The main challenges in using recombinant TRAIL‐based cancer therapy strategies include the inability to treat CRCs caused by TRAIL‐resistant cells. Moreover, another drawback of the recombinant TRAIL protein as a cancer therapy is its short half‐life in the body.^52^, ^53^, ^54^ In TRAIL‐resistant cancer cells, the DR pathway is often compromised because of various factors, such as the downregulation or loss of expression of DR4/DR5 or the overexpression of anti‐apoptotic proteins like c‐FLIP.^55^ However, NDV infection has been shown to overcome TRAIL resistance in HT‐29 CRCs by upregulating the expression of DR4/DR5 and downregulating c‐FLIP expression. TRAIL‐resistant HT‐29 cells typically exhibit low DR5 expression on their cell surface, leading to downregulation of the extrinsic apoptosis signaling initiated by soluble TRAIL binding. The reasons for low DR5 expression in HT‐29 cells are not fully understood, but Kim et al.^56^ have suggested that the overexpression of lipocalin 2, an oncogene, could potentially inhibit DR5 expression. In this study, an rNDV carrying the human TRAIL gene was constructed and tested for its efficacy in TRAIL‐resistant CRC cells, specifically targeting HT‐29 cells that showed low responsiveness to the recombinant TRAIL protein‐based therapy. Therefore, cancer cells exhibiting TRAIL resistance, resulting from reduced DR expression, can have their DR expression enhanced through NDV infection. Additionally, artificially inducing TRAIL protein expression can further enhance the cancer cell‐killing effect. A previous study showed the induction of TRAIL protein expression through the activation of interferon and NF‐κB pathways following viral infection. Moreover, the binding of NDV HN protein to host cells activate TNF and NF‐κB, leading to the transactivation of TNF‐α and TRAIL.^26^ In our study, we observed an increase in TRAIL protein expression in both HCT116 and HT‐29 cells, along with their respective xenograft mouse models, upon rNDV infection. Notably, when rNDV‐TRAIL was administered, TRAIL protein expression increased more strongly (Figure 3A and 5C).

**FIGURE 7:**
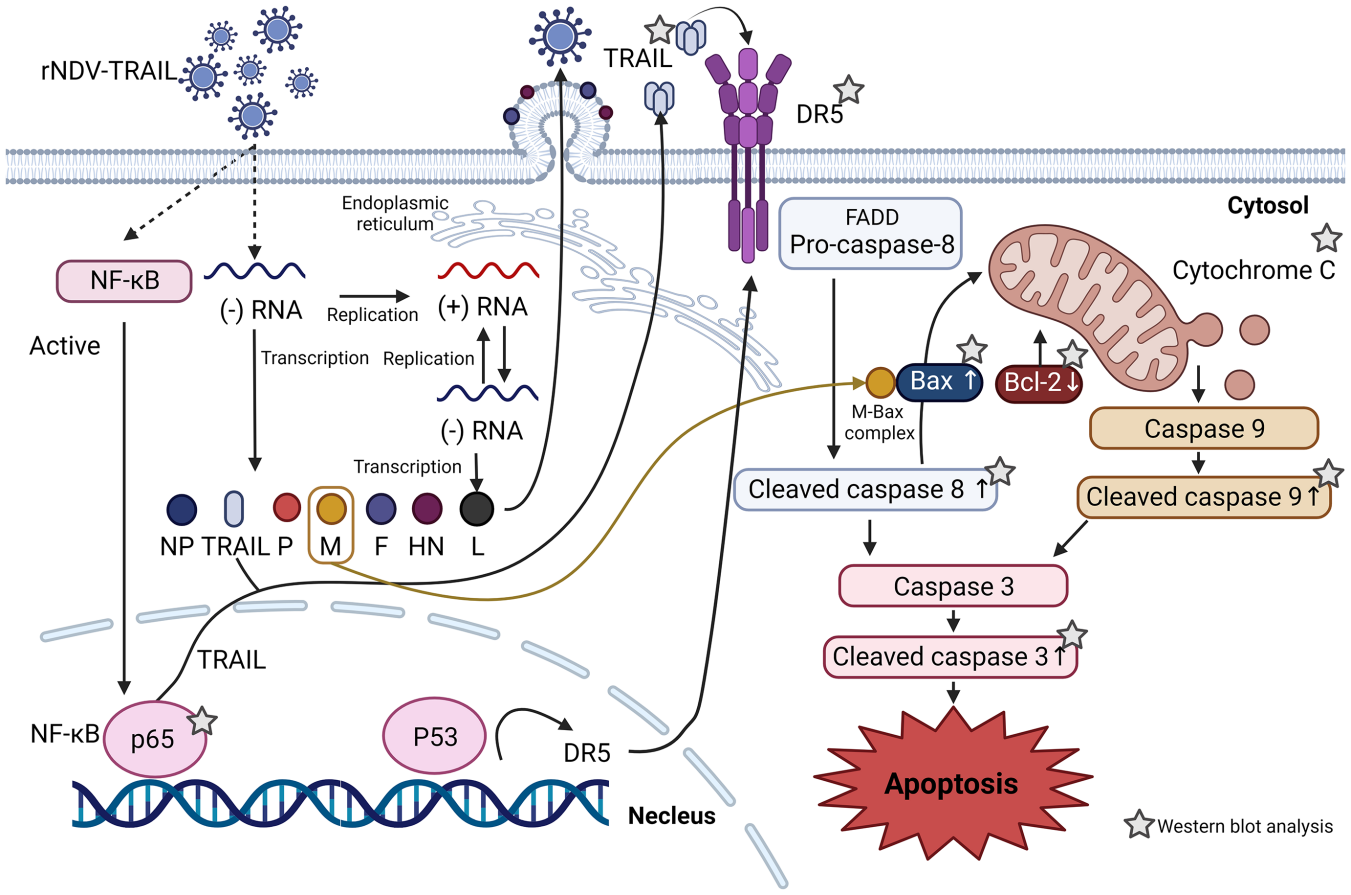
Schematic representation of direct mechanisms of the rNDV‐TRAIL viruses. rNDV containing the TRAIL gene (rNDV‐TRAIL) can stably express the TRAIL gene. Also, rNDV‐TRAIL activates the NF‐кB pathway and induces the secretion of TRAIL. The expressed or induced TRAIL binds to the TRAIL receptor on DR4 or DR5. DR5 promotes TRAIL‐induced apoptotic pathway. The pro‐apoptotic complex is formed. Caspase 8 is cleaved, activates caspase 3, and triggers apoptosis via the extrinsic pathway. Also, caspase 8 induces an intrinsic pathway. The cytochrome C combines with procaspase‐9 to produce apoptosome. Apoptosome triggers caspase 9 followed by the activation of caspase‐3 which leads to ends up to apoptosis.

In the case of TRAIL‐resistant cells HT‐29, rNDV‐TRAIL infection strongly activated caspases‐8, ‐9, and ‐3 as well as the release of cytochrome c from mitochondria, surpassing the effects of rNDV infection alone I. The enhanced expression of DRs, along with theirincreased interaction with TRAIL protein, activated the extrinsic apoptotic pathway. Furthermore, the intrinsic pathway was activated by the increase in BAX protein levels caused by NDV infection. Several protein and cytochrome‐c changes observed in our in vitro experiments were consistently observed in our in vivo experiments, showing similar trends without significant differences (Figure 7).

In this study, we focused on primarily investigating the direct effects of rNDV and rNDV‐TRAIL infection on DR gene expression and apoptotic signaling pathways. While we observed an increase in P‐NF‐κB and Tp53 levels in both HCT116 and HCT‐29 cells after rNDV or rNDV‐TRAIL infection (Figure S3), we did not delve into the detailed investigation of indirect signal transducers or genes that might be involved in DR protein expression. TRAIL DR gene expression is closely related to or dependently controlled by p53 gene activation.^35^ p53 activation is known to promote ferroptosis and influence DR expression.^35^ However, it is important to consider that HT‐29 cells harbor missense mutations in the DNA‐binding domain of p53 (R273H). As a result, the functionality of the mutant P53 protein in altering DR expression may not follow the same pattern as wild‐type P53. Therefore, assuming that a mutant P53 protein might function correctly and alter DR expression would not be a valid hypothesis. In this study, we observed a significant increase in DR expression levels in TRAIL‐resistant CRC cells, specifically HT‐29 cells. The overexpressed TRAIL protein, upon binding to the DR, effectively activated the extrinsic apoptosis pathway in HT‐29 cells. These findings confirmed that rNDV‐TRAIL has the potential to be an exceptionally effective agent for targeting and destroying TRAIL‐resistant cancer cell lines. This innovative approach aims to overcome the limitations of conventional cancer treatments by harnessing the power of recombinant TRAIL protein. By delivering the TRAIL gene into the cytoplasm of cancer cells through rNDV, we successfully achieved the expression of the functional TRAIL protein. The TRAIL protein, which is produced in the cytoplasm of rNDV‐TRAIL‐infected cells, is released into the extracellular environment of the infected cells and binds to DRs on neighboring cancer cells. Subsequently, the apoptotic signal pathway is activated, leading to cancer cell death. In conclusion, the introduction of the TRAIL gene through rNDV infection in cancer cells leads to increased TRAIL protein expression, resulting in enhancer cancer cell death and a potent tumor suppression effect. This innovative approach takes advantage of the ability of artificially expressed TRAIL protein to act on neighboring cancer cells. Notably, the upregulation of DRs caused by rNDV infection, combined with the introduction of the TRAIL gene into cancer cells, leads to enhanced apoptosis and cancer growth inhibition, even in TRAIL‐resistant cancer cells. These findings hold significant promise for potential therapeutic applications. Moving forward, further studies should aim to expand the scope of investigation by testing the efficacy of rNDV containing the TRAIL gene on TRAIL‐resistant cancer cells from various types of cancers.

## AUTHOR CONTRIBUTIONS

**Bo‐Kyoung Jung:** Data curation (lead); investigation (lead); writing – original draft (equal). **Yong Hee An:** Data curation (supporting); investigation (equal). **Sung Hoon Jang:** Data curation (supporting); investigation (supporting). **Gyoungah Ryu:** Investigation (supporting). **Saet‐byel Jung:** Data curation (supporting). **Seonhee Kim:** Investigation (supporting). **Cuk‐Seong Kim:** Data curation (supporting). **Hyun Jang:** Conceptualization (lead); project administration (lead); supervision (lead); writing – review and editing (lead).

## FUNDING INFORMATION

This work was supported by the Technology development Program (S3271268) funded by the Ministry of SMEs and Startups (MSS, Korea).

## CONFLICT OF INTEREST STATEMENT

The authors declare no conflict of interest.

## ETHICS STATEMENT

Approval of the research protocol by an Institutional Reviewer Board: N/A

## ANIMAL STUDIES

This statement is located in Materials and Methods.

## Supporting information


Figure S1.

Figure S2.

Figure S3.

Figure S3A.

Figure S3B.

Figure S3C.

Figure S5A.

Figure S5B.

Figure S5C.

## Data Availability

The data that support the findings of this study are available from the corresponding author upon reasonable request.

## References

[1] Xie J , Itzkowitz SH . Cancer in inflammatory bowel disease. World J Gastroenterol. 2008;14(3):378‐389. 18200660 10.3748/wjg.14.378 PMC2679126

[2] Guinney J , Dienstmann R , Wang X , et al. The consensus molecular subtypes of colorectal cancer. Nat Med. 2015;21(11):1350‐1356. 26457759 10.1038/nm.3967 PMC4636487

[3] Malki A , ElRuz RA , Gupta I , Allouch A , Vranic S , Al Moustafa AE . Molecular mechanisms of colon cancer progression and metastasis: recent insights and advancements. Int J Mol Sci. 2020;22(1):130. 33374459 10.3390/ijms22010130 PMC7794761

[4] Hong SW , Byeon JS . Endoscopic diagnosis and treatment of early colorectal cancer. Intest Res. 2022;20(3):281‐290. 35916019 10.5217/ir.2021.00169 PMC9344247

[5] Ferlay J , Colombet M , Soerjomataram I , et al. Estimating the global cancer incidence and mortality in 2018: GLOBOCAN sources and methods. Int J Cancer. 2019;144(8):1941‐1953. 30350310 10.1002/ijc.31937

[6] Kelly E , Russell SJ . History of oncolytic viruses: genesis to genetic engineering. Mol Ther. 2007;15(4):651‐659. 17299401 10.1038/sj.mt.6300108

[7] Hu PY , Fan XM , Zhang YN , et al. The limiting factors of oncolytic virus immunotherapy and the approaches to overcome them. Appl Microbiol Biotechnol. 2020;104(19):8231‐8242. 32816087 10.1007/s00253-020-10802-w

[8] Reale A , Vitiello A , Conciatori V , Parolin C , Calistri A , Palu G . Perspectives on immunotherapy via oncolytic viruses. Infect Agent Cancer. 2019;14:5. 30792754 10.1186/s13027-018-0218-1 PMC6371415

[9] Schirrmacher V , Fournier P . Newcastle disease virus: a promising vector for viral therapy, immune therapy, and gene therapy of cancer. Methods Mol Biol. 2009;542:565‐605. 19565923 10.1007/978-1-59745-561-9_30 PMC7122391

[10] Lorence RM , Roberts MS , O'Neil JD , et al. Phase 1 clinical experience using intravenous administration of PV701, an oncolytic Newcastle disease virus. Curr Cancer Drug Targets. 2007;7(2):157‐167. 17346107 10.2174/156800907780058853

[11] Duan Z , Deng S , Ji X , Zhao J , Yuan C , Gao H . Nuclear localization of Newcastle disease virus matrix protein promotes virus replication by affecting viral RNA synthesis and transcription and inhibiting host cell transcription. Vet Res. 2019;50(1):22. 30894203 10.1186/s13567-019-0640-4 PMC6425612

[12] Csatary LK , Eckhardt S , Bukosza I , et al. Attenuated veterinary virus vaccine for the treatment of cancer. Cancer Detect Prev. 1993;17(6):619‐627. 8275514

[13] Pecora AL , Rizvi N , Cohen GI , et al. Phase I trial of intravenous administration of PV701, an oncolytic virus, in patients with advanced solid cancers. J Clin Oncol. 2002;20(9):2251‐2266. 11980996 10.1200/JCO.2002.08.042

[14] Freeman AI , Zakay‐Rones Z , Gomori JM , et al. Phase I/II trial of intravenous NDV‐HUJ oncolytic virus in recurrent glioblastoma multiforme. Mol Ther. 2006;13(1):221‐228. 16257582 10.1016/j.ymthe.2005.08.016

[15] Sinkovics JG , Horvath JC . Newcastle disease virus (NDV): brief history of its oncolytic strains. J Clin Virol. 2000;16(1):1‐15. 10.1016/s1386-6532(99)00072-4 10680736

[16] Reichard KW , Lorence RM , Cascino CJ , et al. Newcastle disease virus selectively kills human tumor cells. J Surg Res. 1992;52(5):448‐453. 1619912 10.1016/0022-4804(92)90310-v

[17] Phuangsab A , Lorence RM , Reichard KW , Peeples ME , Walter RJ . Newcastle disease virus therapy of human tumor xenografts: antitumor effects of local or systemic administration. Cancer Lett. 2001;172(1):27‐36. 11595126 10.1016/s0304-3835(01)00617-6

[18] He J , Pan Z , Tian G , et al. Newcastle disease virus chimeras expressing the hemagglutinin‐ neuraminidase protein of mesogenic strain exhibits an enhanced anti‐hepatoma efficacy. Virus Res. 2016;2(221):23‐29. 10.1016/j.virusres.2016.04.023 27164362

[19] Ginting TE , Suryatenggara J , Christian S , Mathew G . Proinflammatory response induced by Newcastle disease virus in tumor and normal cells. Oncolytic Virother. 2017;6:21‐30. 28293547 10.2147/OV.S123292 PMC5345992

[20] Liu T , Zhang Y , Cao Y , et al. Optimization of oncolytic effect of Newcastle disease virus Clone30 by selecting sensitive tumor host and constructing more oncolytic viruses. Gene Ther. 2021;28(12):697‐717. 32409746 10.1038/s41434-020-0145-9 PMC8674137

[21] Huang F , Dai C , Zhang Y , Zhao Y , Wang Y , Ru G . Development of molecular mechanisms and their application on oncolytic Newcastle disease virus in cancer therapy. Front Mol Biosci. 2022;9:889403. 35860357 10.3389/fmolb.2022.889403 PMC9289221

[22] Jang SH , Jung BK , An YH , Jang H . The phosphatase and tensin homolog gene inserted between NP and P gene of recombinant new castle disease virus oncolytic effect test to glioblastoma cell and xenograft mouse model. Virol J. 2022;19(1):21. 35093115 10.1186/s12985-022-01746-w PMC8800283

[23] Syed Najmuddin SUF , Amin ZM , Tan SW , et al. Oncolytic effects of the recombinant Newcastle disease virus, rAF‐IL12, against colon cancer cells in vitro and in tumor‐challenged NCr‐Foxn1nu nude mice. PeerJ. 2020;8:e9761. 33354412 10.7717/peerj.9761 PMC7731658

[24] Zamarin D , Martinez‐Sobrido L , Kelly K , et al. Enhancement of oncolytic properties of recombinant Newcastle disease virus through antagonism of cellular innate immune responses. Mol Ther. 2009 Apr;17(4):697‐706. 19209145 10.1038/mt.2008.286 PMC2835121

[25] Rathmell JC , Thompson CB . The central effectors of cell death in the immune system. Annu Rev Immunol. 1999;17:781‐828. 10358774 10.1146/annurev.immunol.17.1.781

[26] Zeng J , Fournier P , Schirrmacher V . Induction of interferon‐alpha and tumor necrosis factor‐related apoptosis‐inducing ligand in human blood mononuclear cells by hemagglutinin‐neuraminidase but not F protein of Newcastle disease virus. Virology. 2002;297(1):19‐30. 12083832 10.1006/viro.2002.1413

[27] Molouki A , Hsu YT , Jahanshiri F , Rosli R , Yusoff K . Newcastle disease virus infection promotes Bax redistribution to mitochondria and cell death in HeLa cells. Intervirology. 2010;53(2):87‐94. 19955813 10.1159/000264198

[28] Havell EA , Fiers W , North RJ . The antitumor function of tumor necrosis factor (TNF), I. Therapeutic action of TNF against an established murine sarcoma is indirect, immunologically dependent, and limited by severe toxicity. J Exp Med. 1988;167(3):1067‐1085. 3351434 10.1084/jem.167.3.1067 PMC2188888

[29] Seki N , Hayakawa Y , Brooks AD , et al. Tumor necrosis factor‐related apoptosis‐inducing ligand‐mediated apoptosis is an important endogenous mechanism for resistance to liver metastases in murine renal cancer. Cancer Res. 2003;63(1):207‐213. 12517799

[30] Ashkenazi A , Pai RC , Fong S , et al. Safety and antitumor activity of recombinant soluble Apo2 ligand. J Clin Invest. 1999;104(2):155‐162. 10411544 10.1172/JCI6926 PMC408479

[31] Walczak H , Miller RE , Ariail K , et al. Tumoricidal activity of tumor necrosis factor‐related apoptosis‐inducing ligand in vivo. Nat Med. 1999;5(2):157‐163. 9930862 10.1038/5517

[32] Klosek M , Mertas A , Krol W , Jaworska D , Szymszal J , Szliszka E . Tumor necrosis factor‐related apoptosis‐inducing ligand‐induced apoptosis in prostate cancer cells after treatment with xanthohumol‐a natural compound present in Humulus lupulus L. Int J Mol Sci. 2016;17(6):837. 27338375 10.3390/ijms17060837 PMC4926371

[33] James B , Griffith T . Tumor necrosis factor‐related apoptosis‐inducing ligand‐induced apoptotic pathways in cancer immunosurveillance: molecular mechanisms and prospects for therapy. Dove Press. 2014;2015(5):1‐10.

[34] Kan X , Yin Y , Song C , et al. Newcastle‐disease‐virus‐induced ferroptosis through nutrient deprivation and ferritinophagy in tumor cells. iScience. 2021;24(8):102837. 34368653 10.1016/j.isci.2021.102837 PMC8326413

[35] Takimoto R , El‐Deiry WS . Wild‐type p53 transactivates the KILLER/DR5 gene through an intronic sequence‐specific DNA‐binding site. Oncogene. 2000;19(14):1735‐1743. 10777207 10.1038/sj.onc.1203489

[36] Jung BK , An YH , Jang JJ , Jeon JH , Jang SH , Jang H . The human ACE‐2 receptor binding domain of SARS‐CoV‐2 express on the viral surface of the Newcastle disease virus as a non‐replicating viral vector vaccine candidate. PloS One. 2022;17(2):e0263684. 35134091 10.1371/journal.pone.0263684 PMC8824364

[37] Liao Y , Wang HX , Mao X , et al. RIP1 is a central signaling protein in regulation of TNF‐alpha/TRAIL mediated apoptosis and necroptosis during Newcastle disease virus infection. Oncotarget. 2017;8(26):43201‐43217. 28591723 10.18632/oncotarget.17970 PMC5522139

[38] Yurchenko KS , Zhou P , Kovner AV , Zavjalov EL , Shestopalova LV , Shestopalov AM . Oncolytic effect of wild‐type Newcastle disease virus isolates in cancer cell lines in vitro and in vivo on xenograft model. PloS One. 2018;13(4):e0195425. 29621357 10.1371/journal.pone.0195425 PMC5886573

[39] Ahlert T , Schirrmacher V . Isolation of a human melanoma adapted Newcastle disease virus mutant with highly selective replication patterns. Cancer Res. 1990;50(18):5962‐5968. 2203523

[40] Zhao W , Zhang Z , Zsak L , Yu Q . P and M gene junction is the optimal insertion site in Newcastle disease virus vaccine vector for foreign gene expression. J Gen Virol. 2015;96(Pt 1):40‐45. 25274858 10.1099/vir.0.068437-0

[41] Pan Z , He J , Rasoul LM , et al. Identification of optimal insertion site in recombinant Newcastle disease virus (rNDV) vector expressing foreign gene to enhance its anti‐tumor effect. PloS One. 2016;11(10):e0164723. 27736965 10.1371/journal.pone.0164723 PMC5087999

[42] Wu H , Lozano G . NF‐kappa B activation of p53. A potential mechanism for suppressing cell growth in response to stress. J Biol Chem. 1994;269(31):20067‐20074. 8051093

[43] Formentini L , Moroni F , Chiarugi A . Detection and pharmacological modulation of nicotinamide mononucleotide (NMN) in vitro and in vivo. Biochem Pharmacol. 2009;77(10):1612‐1620. 19426698 10.1016/j.bcp.2009.02.017

[44] Cuadrado‐Castano S , Sanchez‐Aparicio MT , Garcia‐Sastre A , Villar E . The therapeutic effect of death: Newcastle disease virus and its antitumor potential. Virus Res. 2015;2(209):56‐66. 10.1016/j.virusres.2015.07.001 PMC4630136 26221764

[45] Chan LC , Kalyanasundram J , Leong SW , et al. Persistent Newcastle disease virus infection in bladder cancer cells is associated with putative pro‐survival and anti‐viral transcriptomic changes. BMC Cancer. 2021;21(1):625. 34044804 10.1186/s12885-021-08345-y PMC8161962

[46] Molouki A , Yusoff K . NDV‐induced apoptosis in absence of Bax; evidence of involvement of apoptotic proteins upstream of mitochondria. Virol J. 2012;30(9):179. 10.1186/1743-422X-9-179 PMC3492152 22935147

[47] Molouki A , Hsu YT , Jahanshiri F , Abdullah S , Rosli R , Yusoff K . The matrix (M) protein of Newcastle disease virus binds to human bax through its BH3 domain. Virol J. 2011;3(8):385. 10.1186/1743-422X-8-385 PMC3166938 21810274

[48] Shayan S , Arashkia A , Bahramali G , Abdoli A , Nosrati MSS , Azadmanesh K . Cell type‐specific response of colon cancer tumor cell lines to oncolytic HSV‐1 virotherapy in hypoxia. Cancer Cell Int. 2022;22(1):164. 35477503 10.1186/s12935-022-02564-4 PMC9044800

[49] Park YR , Lee ST , Kim SL , et al. MicroRNA‐9 suppresses cell migration and invasion through downregulation of TM4SF1 in colorectal cancer. Int J Oncol. 2016;48(5):2135‐2143. 26983891 10.3892/ijo.2016.3430

[50] Sun S , Li Z , Sun L , et al. Results on efficacy and safety of cancer treatment with or without tumor necrosis factor‐related apoptosis‐inducing ligand‐related agents: a meta‐analysis. Mol Clin Oncol. 2014;2(3):440‐448. 24772315 10.3892/mco.2014.261 PMC3999132

[51] Soria JC , Mark Z , Zatloukal P , et al. Randomized phase II study of dulanermin in combination with paclitaxel, carboplatin, and bevacizumab in advanced non‐small‐cell lung cancer. J Clin Oncol. 2011;29(33):4442‐4451. 22010015 10.1200/JCO.2011.37.2623

[52] Herbst RS , Eckhardt SG , Kurzrock R , et al. Phase I dose‐escalation study of recombinant human Apo2L/TRAIL, a dual proapoptotic receptor agonist, in patients with advanced cancer. J Clin Oncol. 2010;28(17):2839‐2846. 20458040 10.1200/JCO.2009.25.1991

[53] Soria JC , Smit E , Khayat D , et al. Phase 1b study of dulanermin (recombinant human Apo2L/TRAIL) in combination with paclitaxel, carboplatin, and bevacizumab in patients with advanced non‐squamous non‐small‐cell lung cancer. J Clin Oncol. 2010;28(9):1527‐1533. 20159815 10.1200/JCO.2009.25.4847

[54] Wu X , Wang S , Li M , et al. Nanocarriers for TRAIL delivery: driving TRAIL back on track for cancer therapy. Nanoscale. 2017;9(37):13879‐13904. 28914952 10.1039/c7nr04959e

[55] Zhou X , Jiang W , Liu Z , Liu S , Liang X . Virus infection and death receptor‐mediated apoptosis. Viruses. 2017;9(11):316. 29077026 10.3390/v9110316 PMC5707523

[56] Kim SL , Min IS , Park YR , Lee ST , Kim SW . Lipocalin 2 inversely regulates TRAIL sensitivity through p38 MAPK‐mediated DR5 regulation in colorectal cancer. Int J Oncol. 2018;53(6):2789‐2799. 30221676 10.3892/ijo.2018.4562

