# Supplementary figures and images for: The tumor suppressive effect and apoptotic mechanism of TRAIL gene‐containing recombinant NDV in TRAIL‐resistant colorectal cancer HT‐29 cells and TRAIL‐nonresistant HCT116 cells, with each cell bearing a mouse model
Source: Cancer Med. 2023 Oct 16;12(20):20380–95. doi: 10.1002/cam4.6622 (PMC10652305; doi:10.1002/cam4.6622)

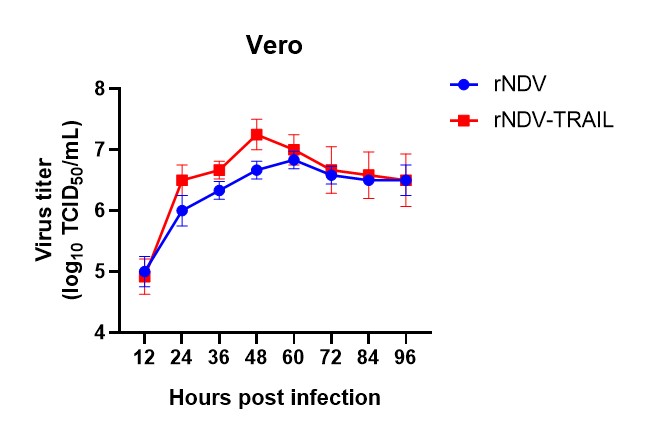

Supplement: Supplementary file 1 — Figure S1. [file CAM4-12-20380-s001.jpg]

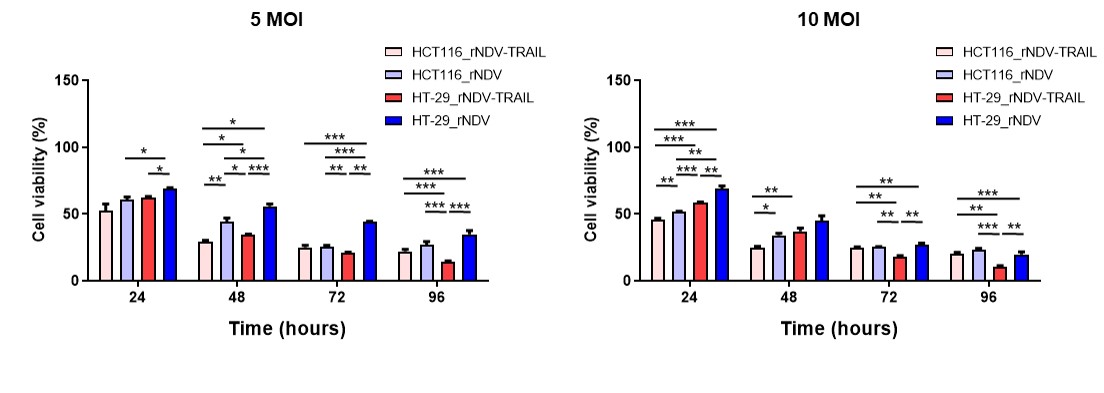

Supplement: Supplementary file 2 — Figure S2. [file CAM4-12-20380-s005.jpg]

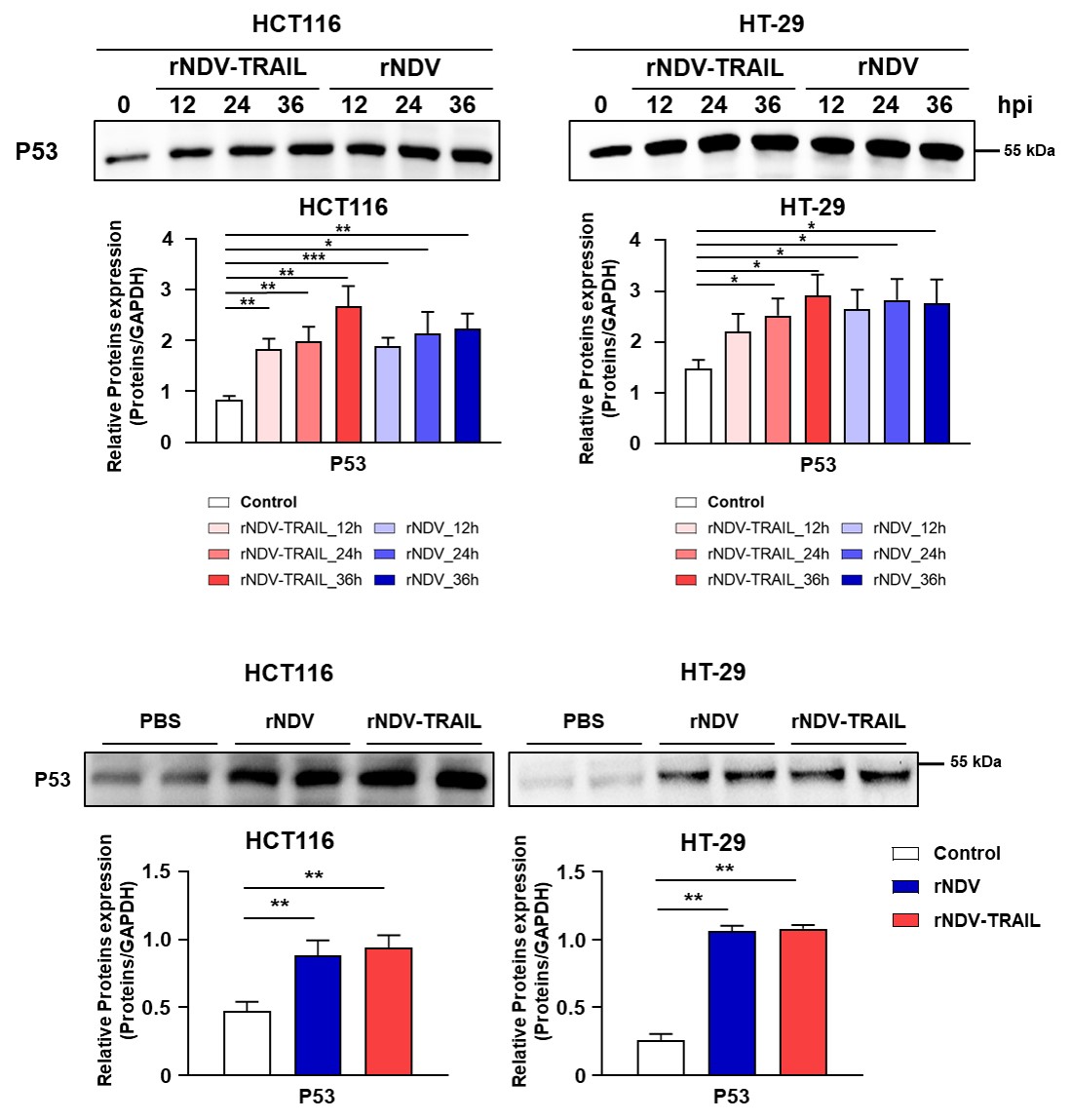

Supplement: Supplementary file 3 — Figure S3. [file CAM4-12-20380-s007.jpg]

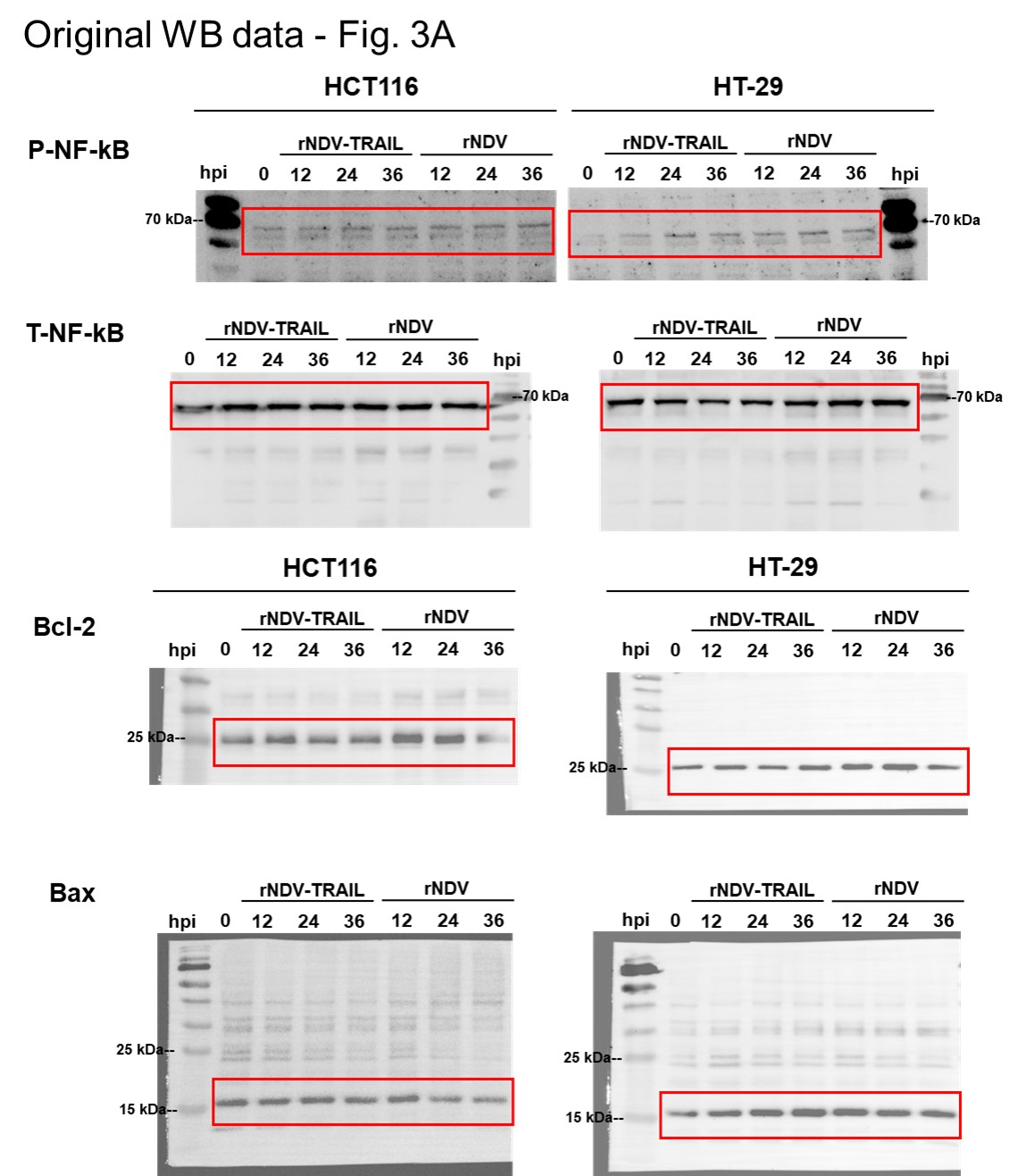

Supplement: Supplementary file 4 — Figure S3A. [file CAM4-12-20380-s008.jpg]

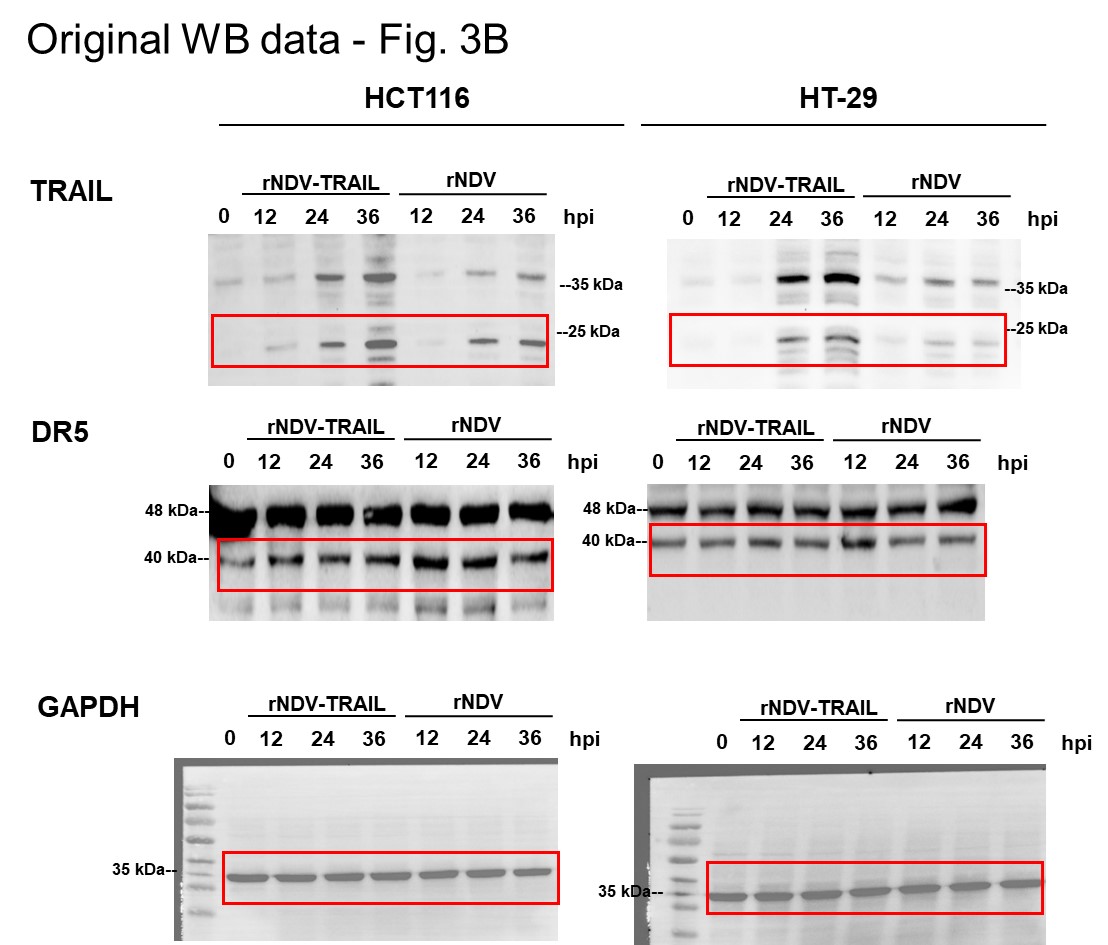

Supplement: Supplementary file 5 — Figure S3B. [file CAM4-12-20380-s006.jpg]

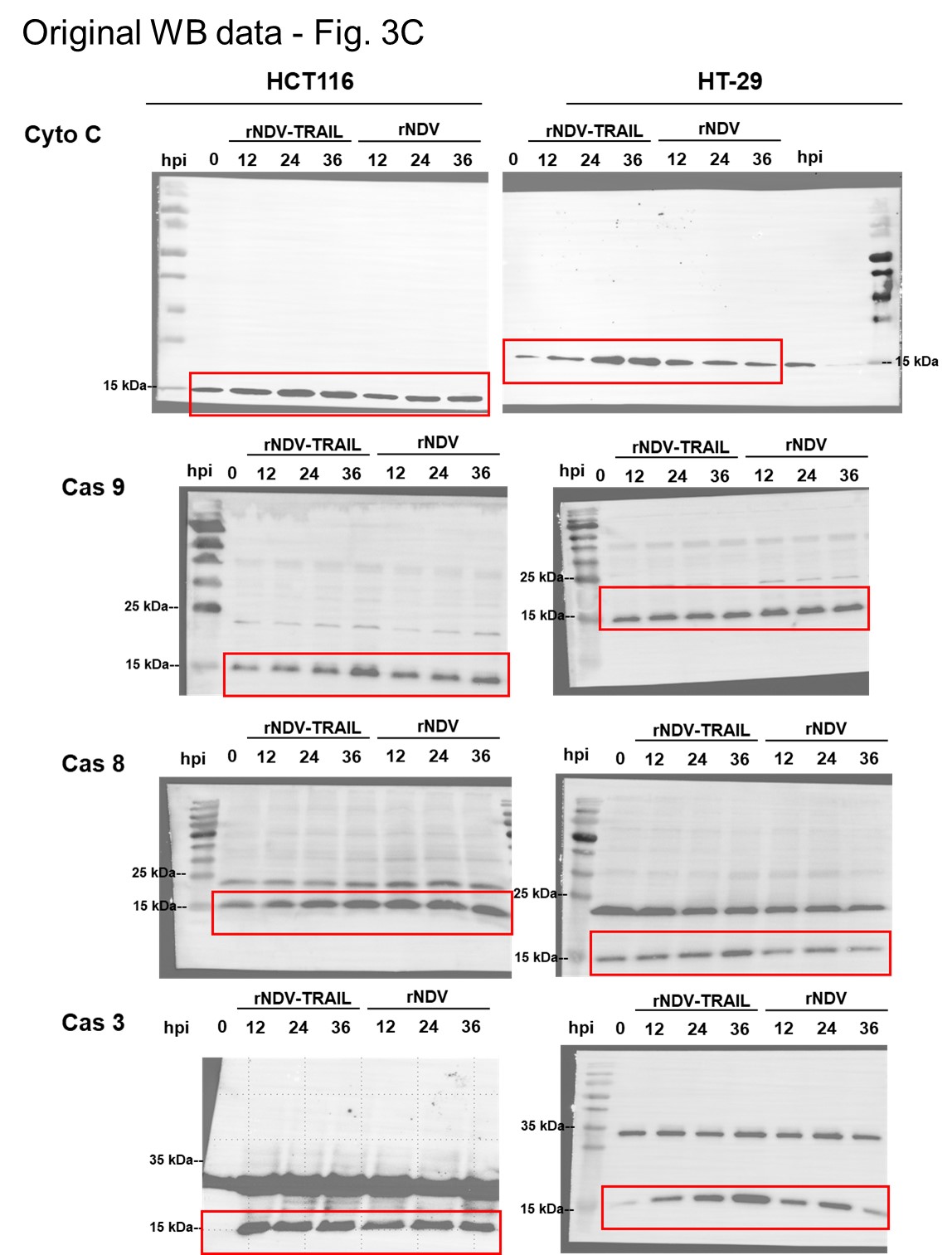

Supplement: Supplementary file 6 — Figure S3C. [file CAM4-12-20380-s002.jpg]

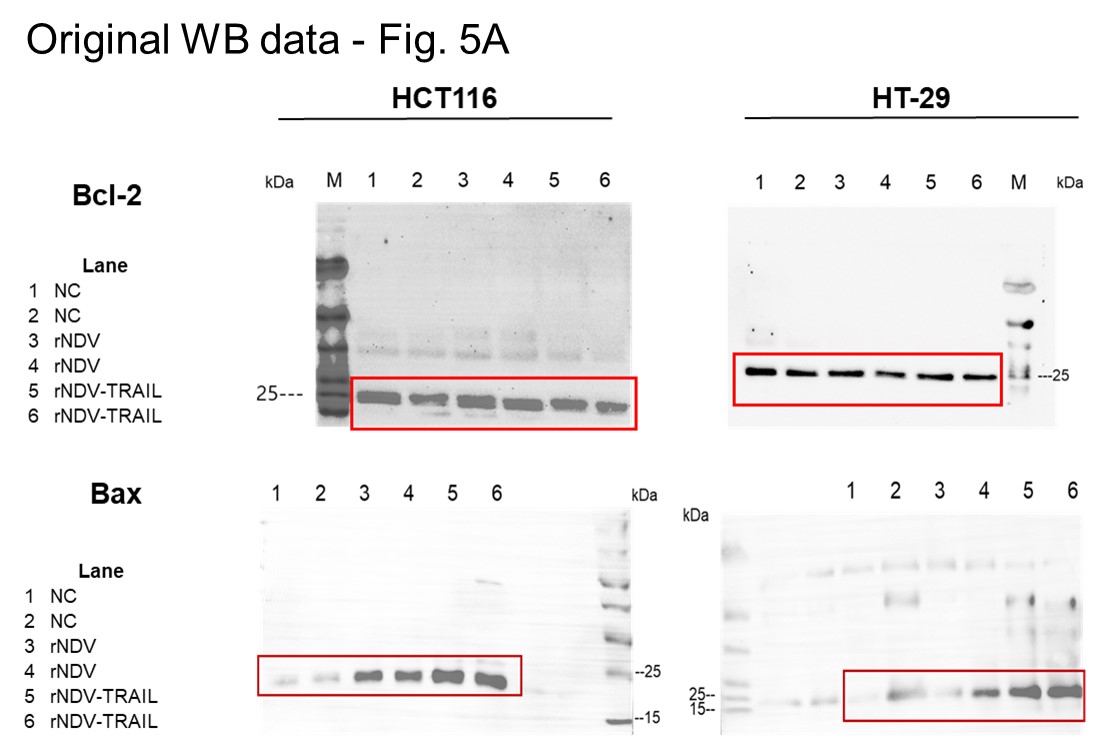

Supplement: Supplementary file 7 — Figure S5A. [file CAM4-12-20380-s003.jpg]

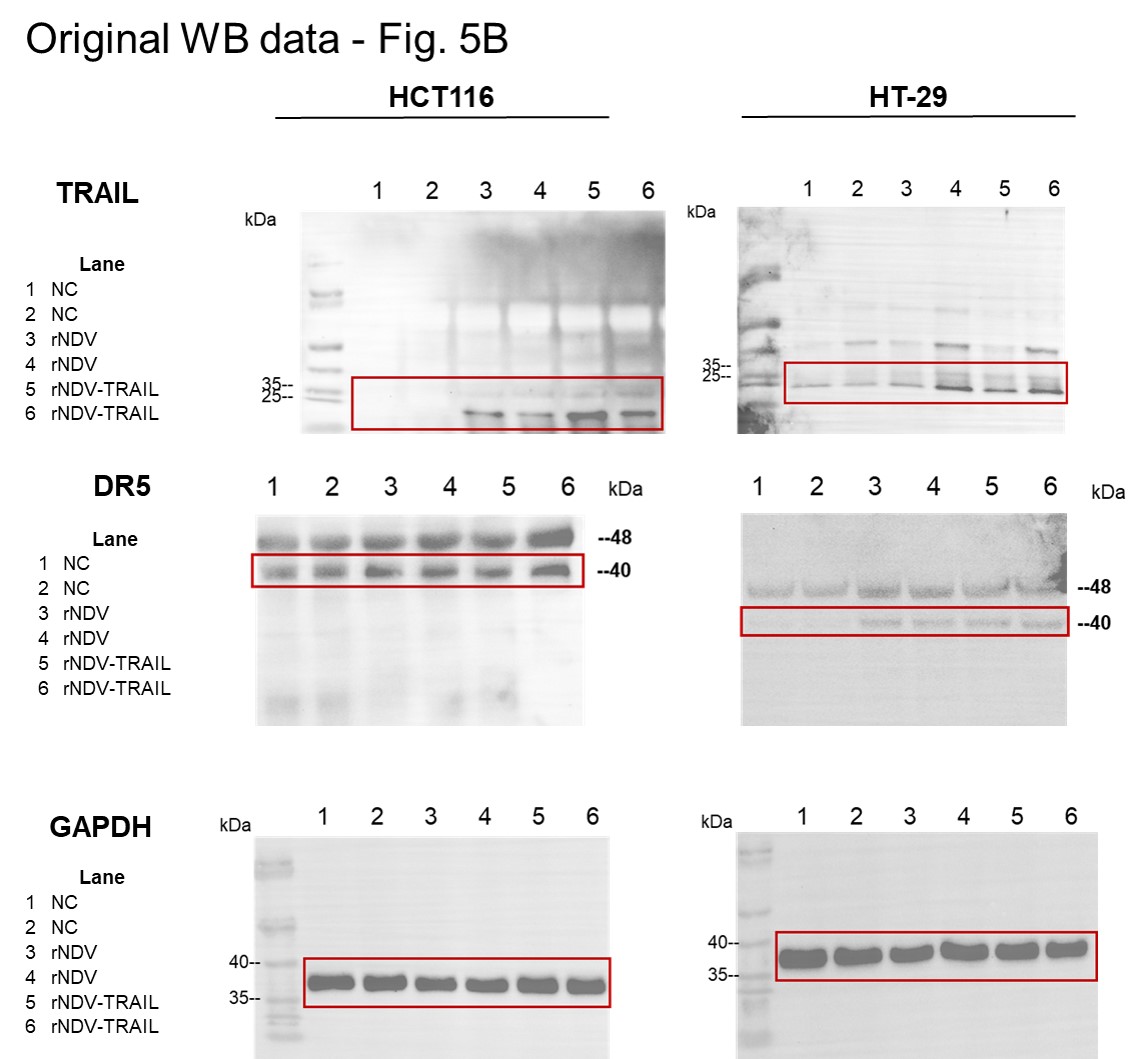

Supplement: Supplementary file 8 — Figure S5B. [file CAM4-12-20380-s009.jpg]

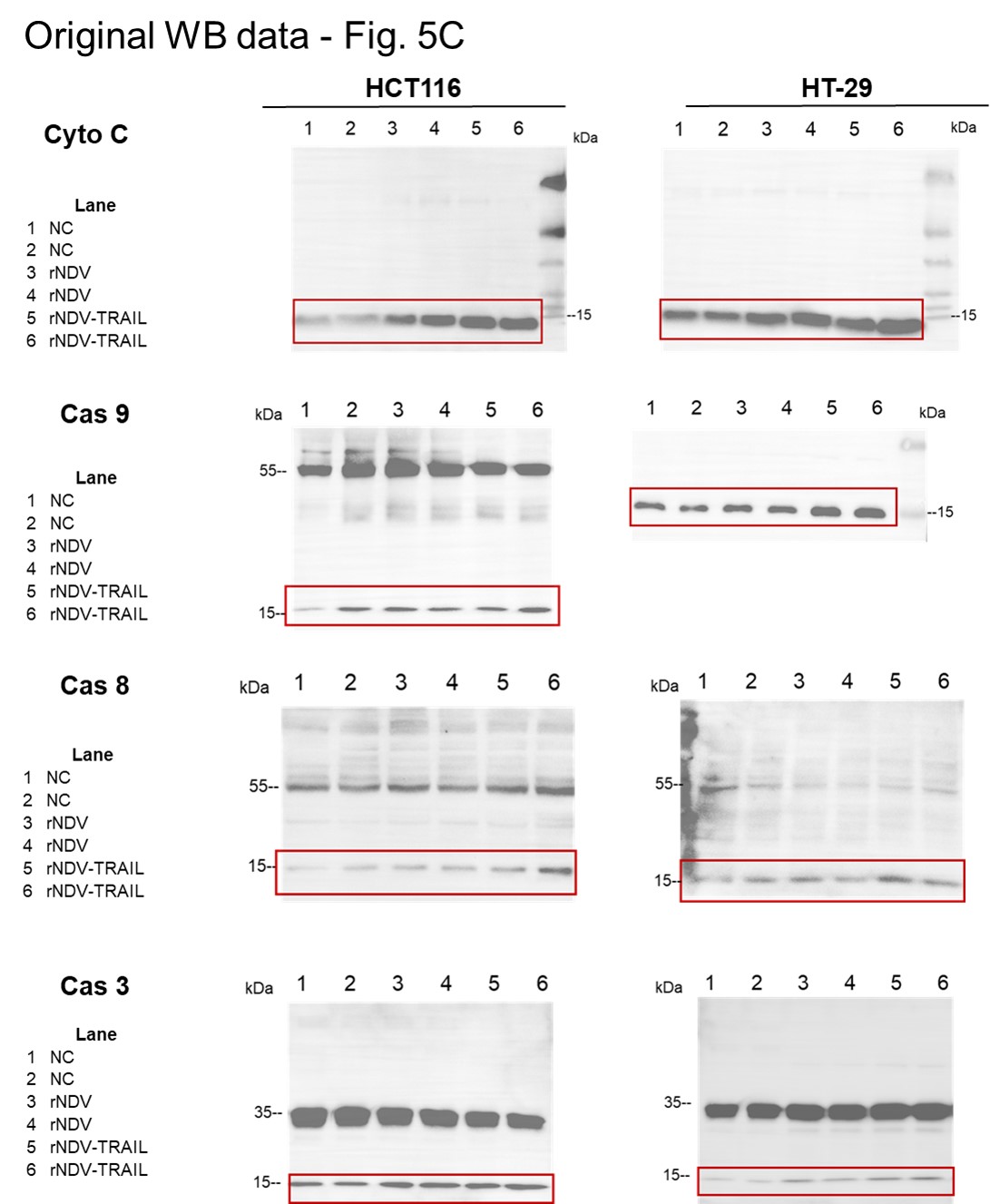

Supplement: Supplementary file 9 — Figure S5C. [file CAM4-12-20380-s004.jpg]
